# HNRNPC Succinylation Influences the Neurodegeneration of Alzheimer's Disease Through YME1L1‐Mediated Mitochondrial Metabolism

**DOI:** 10.1111/acel.70646

**Published:** 2026-08-05

**Authors:** Xuewei Li, Fan Yang, Yuyan Jiang, Fei Zhao, Fan Liu

**Affiliations:** ^1^ Department of Neurology, The First Affiliated Hospital Hengyang Medical School, University of South China Hengyang Hunan China; ^2^ Department of Neurology, The Second Affiliated Hospital Hengyang Medical School, University of South China Hengyang Hunan China

**Keywords:** Alzheimer's disease, HNRNPC, mitochondrial metabolism, SIRT5, YME1L1

## Abstract

Mitochondrial dysfunction and abnormal energy metabolism are important pathological features of Alzheimer's disease (AD). This study investigates how mitochondrial protease YME1L1 affects mitochondrial function and its upstream regulation in the pathogenesis of AD. The AD model was established by using APP/PS1 transgenic mice, primary neurons treated with Aβ1‐42, and HT22 cells. The silencing of YME1L1 was achieved to evaluate its effects on mitochondrial function and OPA1 protein hydrolysis. RIP‐qPCR and RNA pull‐down test were used to evaluate the interaction between HNRNPC and YME1L1 mRNA. The protein succinylation level was detected by proteomic analysis of succinylation, and co‐immunoprecipitation (Co‐IP) was used to verify the succinylation of HNRNPC. Cognitive ability was tested by behavioral tests, including the Morris water maze, Y‐maze, object recognition test, and olfactory test. Finally, the therapeutic potential of SIRT5 was studied by an overexpression experiment in an AD model. YME1L1 was significantly upregulated in the AD model, which promoted mitochondrial dysfunction and neuronal damage through OPA1 hydrolysis. HNRNPC enhances the stability of YME1L1 mRNA through an m6A‐dependent mechanism, while its own K50 succinylation enhances the stability of HNRNPC by competitively inhibiting TRIM25‐mediated ubiquitination, further amplifying the expression of YME1L1. SIRT5 downregulation in AD elevated HNRNPC succinylation levels. SIRT5 overexpression promoted HNRNPC desuccinylation, reduced YME1L1 expression, restored mitochondrial function, and ameliorated Aβ deposition and cognitive deficits in AD mice. The SIRT5‐HNRNPC‐YME1L1 axis contributes to AD pathogenesis by disrupting OPA1 proteolysis and mitochondrial dynamics. Targeting HNRNPC succinylation represents a promising therapeutic strategy for AD.

## Graphical abstract

In Alzheimer's disease, SIRT5 downregulation enhances TRIM25‐mediated succinylation of HNRNPC at K50, increasing its stability. Stabilized HNRNPC upregulates YME1L1 expression via an m6A‐dependent mechanism, leading to OPA1 hydrolysis dysregulation, mitochondrial fission‐fusion imbalance, ROS overproduction, and neuronal death.

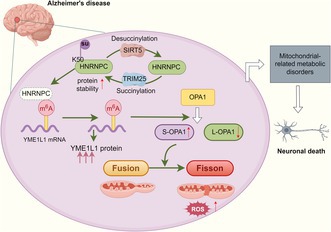

**Abbreviations**
AChEIs: acetylcholinesterase inhibitors
ActD: actinomycin D
AD: Alzheimer's disease
APP: amyloid precursor protein
Aβ: amyloid β‐protein
CCK8: cell counting kit‐8
CHX: cycloheximide
Co‐IP: co‐immunoprecipitation
DIA: data independent collection
Drp1: dynamin‐related protein 1
Fis1: mitotic protein 1
HFIP: hexafluoro‐2‐propanol
IHC: immunohistochemical
IMM: inner mitochondrial membrane
L‐OPA1: long‐chain OPA1
MFN1/2: mitochondrial fusion protein 1/2
MWM: morris water maze
NMDA: N‐methyl‐D‐aspartic acid
OMM: outer mitochondrial membrane
OPA1: optic atrophy‐related protein 1
PCR: polymerase chain reaction
PTMs: posttranslational modifications
RIP‐qPCR: RNA immunoprecipitation qPCR
ROS: reactive oxygen species
SAP: spontaneous alternating representation
SIRT: silent information regulators
S‐OPA1: short‐chain OPA1
TEM: transmission Electron Microscope
WB: western blot
WT: wild‐type
YME1L1: YME1‐Like 1 ATPase

## 1 Introduction

About 60%–70% of dementia cases are caused by Alzheimer's disease (AD), an age‐related neurodegenerative illness. It is now the seventh greatest cause of death globally and mostly results in declines in thinking, behavior, language, and memory (“2024 Alzheimer's Disease Facts and Figures” 2024). The pathogenic mechanism of AD remains incompletely understood, with prevailing theories including the amyloid β‐protein (Aβ) hypothesis, the tau protein dysregulation theory, and neurotransmitter abnormalities (Guo et al. 2020). Currently, only a limited number of drugs approved for treating AD are used clinically, such as acetylcholinesterase inhibitors (AChEIs), N‐methyl‐D‐aspartic acid (NMDA) receptor antagonists, and Aβ‐targeting drugs. However, the effects of these medications are limited to alleviating certain symptoms in AD patients and cannot alter the progression of the disease (Sugandhi et al. 2025).

Mitochondria are the primary sites for the generation of oxygen‐free radicals, which fuel cellular respiration. Additionally, mitochondria are crucial for various metabolic processes, including the metabolism of reactive oxygen species (ROS) and calcium homeostasis (Glover et al. 2024). According to research, one of the first pathogenic changes in AD is mitochondrial dysfunction (Reddy and Oliver 2019). Generally, the mitochondrial antioxidant system detoxifies ROS through various mechanisms and maintains a balance between harmful free radical production and antioxidant protection. Therefore, once the imbalance occurs, oxidative stress increases, the macromolecules in the mitochondrial structure are vulnerable to oxidative damage, and the function of organelles is destroyed, which leads to mtDNA mutation (Kung et al. 2021), which in turn damages the mitochondrial function of nerve cells and is easy to evolve into AD. Moreover, heightened expression of genes governing mitochondrial energy metabolism results in cellular metabolic dysfunction in AD. This dysregulation promotes the creation and aggregation of tau and Aβ proteins, worsening mitochondrial dysfunction and perpetuating a detrimental cycle (Reddy and Oliver 2019). Our hypothesis suggests that intervention in mitochondrial homeostasis to restore normal mitochondrial function and energy metabolism can provide valuable therapeutic strategies for AD.

Mitochondria control their quantity and shape by an ongoing cycle of fusion and fission, which makes it easier for their contents to swap places in response to the cell's energy and metabolic needs (Chan 2020). Mitochondrial fusion is driven by Mitochondrial Fusion Protein 1/2 (MFN1/2) on the outer mitochondrial membrane (OMM) and Optic Atrophy‐related Protein 1 (OPA1) on the inner mitochondrial membrane (IMM) (Yang, Wang, et al. 2022), while mitotic division is coordinated by Dynamin‐related Protein 1 (Drp1), Mitochondrial Fission Protein 1 (Fis1), and mitochondrial fission factors. A delicate equilibrium between fission and fusion maintains mitochondrial self‐renewal (Finocchietto et al. 2022; Wang et al. 2024). The function of OPA1 is regulated by proteolytic processing at the S1 and S2 cleavage sites by overlapping activity with m‐AAA protease (OMA1) and YME1‐like 1 ATPase (YME1L1) (Anand et al. 2014; Wai et al. 2015). Under cellular stress conditions, increased YME1L1 activity initiates the cleavage of long‐chain OPA1 (L‐OPA1) into short‐chain OPA1 (S‐OPA1), thereby inducing mitochondrial fission and disrupting cristae morphology (Baker et al. 2014). Increasing evidence indicates that abnormal mitochondrial dynamics are implicated in various pathological processes, including AD (Oliver et al. 2019). Therefore, maintaining the balance between L‐OPA1 and S‐OPA1 is crucial for regulating mitochondrial dynamics in AD (Ahmed et al. 2019; Wai et al. 2015). Notably, Beck (2016) reported that YME1L1 mRNA levels were significantly upregulated by approximately 40%–60% in the frontal cortex of sporadic AD patients and by approximately 70%–90% in familial AD patients. Consistent with this finding, our preliminary bioinformatics analysis also revealed significantly elevated YME1L1 expression levels in the brain tissue of AD patients. However, the upstream regulatory mechanisms of YME1L1 expression in AD remain unclear.

Mitochondrial dysfunction is closely associated with posttranslational modifications (PTMs) of proteins (S. Wang et al. 2022). Lysine succinylation (Ksucc) is a new PTM, which regulates mitochondrial function by participating in glycolysis, lipid metabolism, tricarboxylic acid cycle, and ketone body metabolism (F. Wang et al. 2017; Yuan et al. 2022). Elevated succinylation, accompanied by disrupted neuronal transcriptional regulation, may affect neuronal pathways directly involved in the pathogenesis of brain disease (Lancaster et al. 2023). Studies indicate that abnormal succinylation modifications are closely associated with the onset of AD (Yang, Tapias, et al. 2022). Quantitative analysis of the brain protein group of AD patients showed that succinylation levels of various mitochondrial proteins decreased, while succinylation levels of amyloid precursor protein (APP) and tau protein increased (Yuan et al. 2022), thus promoting Aβ accumulation and plaque formation. In addition, the succinylation modification of protein is regulated by succinyltransferase and dessuccinylating enzyme. It is worth noting that the family of silent information regulators (SIRT), including SIRT5/7, can remove Ksucc modification from mitochondrial function‐related proteins, thus maintaining stable mitochondrial function and preventing disease onset (Swerdlow 2017; Yuan et al. 2022). There is still much to learn about the precise function of SIRT5/7 in AD and whether it controls the production of proteins linked to mitochondrial function by desuccinylation modification.

Accumulating evidence has confirmed that mitochondrial dysfunction is a key pathological feature of AD (Song et al. 2021), and succinylation has been demonstrated to regulate mitochondrial function (Wang et al. 2017; Yuan et al. 2022), with its abnormal modification closely associated with AD pathogenesis (Yang, Tapias, et al. 2022). However, the relationship between succinylation and neuronal mitochondrial dynamics in AD remains unclear. We speculate that succinylation may influence mitochondrial dynamics by regulating the activity or stability of mitochondrial function‐related proteins. Given that YME1L1 is a key protease involved in maintaining mitochondrial homeostasis, we hypothesize that succinylation may regulate YME1L1 expression or function, thereby contributing to mitochondrial dynamics imbalance and neurodegeneration in AD. Therefore, this study aims to investigate the expression changes of YME1L1 in AD and its role in maintaining mitochondrial homeostasis, and to determine whether succinylation regulates YME1L1 expression or function, thereby participating in AD pathogenesis. We found that YME1L1 expression is significantly upregulated in the AD model, and its knockdown can improve mitochondrial dysfunction and cognitive impairment. Further mechanistic studies indicate that HNRNPC stabilizes YME1L1 mRNA through an m6A‐dependent mechanism, and its K50 succinylation enhances its own stability by inhibiting TRIM25‐mediated ubiquitination, while the downregulation of SIRT5 in AD increases the succinylation level of HNRNPC at the K50 site, thereby enhancing HNRNPC stability and subsequently promoting YME1L1 expression. We propose that the SIRT5‐HNRNPC‐YME1L1 regulatory axis represents a key pathway underlying mitochondrial dysfunction and neurodegenerative lesions in AD and may serve as a novel therapeutic target for AD treatment.

## 2 Materials and Methods

### 2.1 Animals

Male APP/PS1 mice and adult C57BL/6J mice (6 months old) were purchased from Hunan SJA Laboratory Animal. After a week's adaptation period, the formal experimental procedure began. C57BL/6J mice served as the wild‐type (WT) control group, while APP/PS1 mice served as the spontaneous AD model. APP/PS1 mice in the APP/PS1+sh‐NC, APP/PS1+sh‐YME1L1–1, APP/PS1+sh‐YME1L1–2, LV‐NC, LV‐SIRT5, and LV‐SIRT5+LV‐HNRNPC groups received stereotaxic injections of corresponding lentiviral vectors (LV‐sh‐NC, LV‐sh‐YME1L1–1, LV‐sh‐YME1L1–2, LV‐SIRT5 and LV‐HNRNPC) into the hippocampal CA1 region at the anterior fontanelle −2.0 mm, the middle‐lateral 1.0 mm, and the depth 1.5 mm. The injection volume was 2 μL (10^12^–10^13^ v.g./mL), administered at a rate of 0.2 μL/min. A series of behavioral tests was conducted 3 weeks post‐viral injection, with tissue collection taking place upon the conclusion of the behavioral assessments. The study adhered to the National Institutes of Health guidelines for Laboratory Animal Care and Use and was approved by the Medical Ethics Review Committee of the First Affiliated Hospital of the University of South China (Approval No. 2025LL0401001).

### 2.2 Cell Culture and Treatment

First, primary neurons were isolated, and a cell culture was performed. After disinfecting suckling mice from C57BL/6J mice within 24 h of birth, the cerebral cortex was isolated in a laminar flow hood. Tissue fragments were digested with trypsin, resuspended, filtered, and centrifuged to obtain a pellet. The pellet was resuspended in neuron‐specific culture medium (iCell‐0013‐500 mL; iCell, China) and seeded into poly‐L‐lysine‐coated 96‐well plates. Cells were cultured for 7 days prior to experimentation. The mouse hippocampal neuronal cell line HT22 (AW‐CNM116; Abiowell, China) was cultured in a 37°C incubator with 5% CO_2_.

And then the cells were processed as follows. Primary neurons and HT22 cells were transfected with different plasmids for 6 h, including sh‐YME1L1 (HG‐SH013771), sh‐NC, sh‐HNRNPC (HG‐SH016884), WT‐HNRNPC (HG‐MO016884), HNRNPC‐K50R (HG‐MO016884M), oe‐YME1L1 (HG‐MO013771), oe‐SIRT5 (HG‐MO178848), and oe‐SIRT5‐H158Y (HG‐MO178848M). For S‐OPA1 overexpression, primary neurons were transduced with LV‐S‐OPA1 lentivirus for 48 h. YME1L1‐1 siRNA sequence: 5′‐GTCAATACACCTGTTTCTCAA‐3′; YME1L1‐2 siRNA sequence: 5′‐AGGCAACATCCAAAAACTCTT‐3′; HNRNPC‐1 siRNA sequence: 5′‐ACCCTGTAAGTTTTGCTTTAA‐3′; HNRNPC‐2 siRNA sequence: 5′‐GACTTAACAATAAAAAGTTGT‐3′. After transfection, Aβ‐induced mouse hippocampal primary neurons and HT22 cells were used to establish cellular models of AD. Solid Aβ peptide (P9001, Beyotime, China) was dissolved in cold hexafluoro‐2‐propanol (HFIP) (105228, Sigma, USA), then allowed to evaporate to dryness in a fume hood. The resulting film was dissolved in anhydrous DMSO at a concentration of 5 mM, followed by vortex dilution to the appropriate concentration in buffer (ice‐cold PBS/serum‐free, phenol red‐free DMEM/F12 medium). The sample was then centrifuged at 14,000 *g* for 10 min at 4°C–8°C; soluble oligomers were present in the supernatant. The supernatant was diluted for use in experiments. Primary neurons or HT22 cells were cultured for 48 h in neurotrophic medium containing 5 μM Aβ1‐42 or an interference control peptide. Each treatment was replicated in triplicate.

### 2.3 Quantitative Reverse Transcription‐Polymerase Chain Reaction (PCR)

Total RNA was extracted from tissues using the Trizol (15596026; Thermo, USA) method, followed by chloroform fractionation, isopropanol precipitation, washing, and dissolution. RNA was reverse transcribed into cDNA using a reverse transcription kit (CW2569; CWbio, China). Using the cDNA as a template, real time quantitative PCR was performed using the SYBR Green method. Specific primers were employed to amplify the target genes, with β‐actin serving as the internal control. Each sample was tested in triplicate. The reaction program included pre‐denaturation, amplification, and melting curve analysis. Primer sequences are listed in Table 1.

**TABLE 1:** Primer sequence.

Primers	Sequence	Product length/bp
M‐actin	F ACATCCGTAAAGACCTCTATGCC R TACTCCTGCTTGCTGATCCAC	223
M‐YME1L1	F ATGCACCCGTATTCAAGGCA R TTCGACCCTTCACATCTGGC	184
M‐Hnrnpc	F ACAGAGACAGCGCCAA R TTCATCTCCCACAAACGCCTA	100

### 2.4 Western Blot (WB)

SDS‐PAGE electrophoresis was performed using a 10% separating gel and a 4.8% concentrating gel, and 10 μg of protein was loaded into each well. After electrophoresis, the membrane was transferred to the Nitrocellulose Membrane under a constant current of 300 mA. After blocking with 5% skim milk powder (AWB0004; Abiowell, China) for 90 min, the membrane was incubated with the primary antibody (Table 2) at 4°C overnight. After washing, the membrane was incubated with HRP‐conjugated secondary antibody at room temperature for 90 min, and then developed by ECL chemiluminescence (AWB0005; Abiowell, China). The signal was collected in the imaging system.

**TABLE 2:** Antibody information.

Name	Item number	Dilution ratio	Company
YME1L1	ab234744	1:3000	Abcam (UK)
HNRNPC	11760‐1‐AP	1:20000	Proteintech (USA)
SIRT5	15122‐1‐AP	1:8000	Proteintech
SIRT7	AWA12742	1:1000	Abiowell (China)
OPA1	ab157457	1:3000	Abcam
IGF2BP1	22803‐1‐AP	1:20000	Proteintech
YTHDF1	17479‐1‐AP	1:4000	Proteintech
IGF2BP3	14642‐1‐AP	1:5000	Proteintech
YTHDF2	24744–1‐AP	1:5000	Proteintech
YTHDF3	ab220161	1:1000	Abcam
IGF2BP2	11601‐1‐AP	1:8000	Proteintech
OMA1	bs‐19641R	1:1000	Bioss (China)
β‐actin	66,009‐1‐Ig	1:5000	Proteintech
HRP goat anti‐mouse IgG	SA00001‐1	1:6000	Proteintech
HRP goat anti‐rabbit IgG	SA00001‐2	1:6000	Proteintech

### 2.5 Morris Water Maze Test (MWM)

Mice were placed in a circular pool of water and given free access to swim. Throughout each trial, the amount of time needed to get to the platform and the number of platform crossings were noted. Mice were randomly assigned to one of four beginning positions in the water for four trials each day for the first 4 days of training, with a 20‐min break between sessions. A mouse was positioned on the platform for 30 s while it was unable to reach it. Although the mouse successfully located the platform, it was permitted to remain there for 15 s. On the fifth day, after removing the platform, each mouse was put into the pool, and its search for the platform within 120 s was observed and recorded. The escape latency during training, the frequency of crossing the platform position, and the duration in the target quadrant during the fifth day of testing were analyzed to evaluate the learning and memory of mice. All mice were confirmed to have normal visual function by the visual cliff test before the water maze test. Swimming speed and path length were recorded and analyzed to exclude potential confounding effects related to motor function or swimming ability.

### 2.6 Y‐Maze

The Spontaneous Alternating Representation (SAP) test of the Y‐maze evaluates the ability to identify the previously explored environment. This maze consists of three arms (8 × 30 × 15 cm) with an angle of 120 degrees. Put the mouse in the center of the Y‐maze and allow 10 min to explore. In order to keep clean, wipe the arm with 70% ethanol solution between experiments. The calculation method of SAP is the actual number of alternating times divided by (total number of arms‐2) × 100, which represents the percentage of new arms entering continuously in three tests. Total arm entries were recorded to exclude potential confounding effects of locomotor activity.

### 2.7 Object Recognition Test

During the training phase, mice were allowed to explore two identical objects for 10 min. In the testing phase, 1 h after training, each mouse was returned to the box, which had been modified to contain both familiar and novel objects. The box and objects were cleaned before each test to eliminate odor cues. The calculation method of exploration time is the time for each mouse to smell with its nose or point its paw at an object. The “recognition index” refers to the time spent exploring the novel object relative to the time spent exploring both objects. All mice passed the visual cliff test, confirming normal visual function required for object discrimination. Total exploration time was recorded to exclude potential confounding effects of motivation level or locomotor activity.

### 2.8 Olfactory Test

Mice were fasted for 16 h and then placed in cages containing 3 cm of bedding material with three mouthfuls of food (small pieces of peanut butter‐flavored cereal). During the test, each mouse had 5 min to locate the buried food. The latency period for retrieving the buried food was recorded. Individuals performing the tests and analyzing the data were unaware of the mice's genotypes. All mice were confirmed to have normal olfactory function before testing, as they successfully located food within the 5‐min limit.

### 2.9 Immunohistochemical (IHC) Assay

Immunohistochemistry was employed to detect Aβ expression in mouse brain tissue. Tissue sections underwent dewaxing, antigen thermal repair, and endogenous enzyme blocking before incubation with primary antibody anti‐Aβ (AWA10572; Abiowell, China) overnight at 4°C. Subsequently, sections were incubated with HRP‐labeled secondary antibody at 37°C for 30 min, followed by DAB staining, counterstained with hematoxylin, dehydrated and transparent, and sealed. Images were captured and analyzed under a light microscope using mean optical density (IOD) and positive rate for quantitative assessment.

### 2.10 Transmission Electron Microscope (TEM)

The mitochondrial structure of the hippocampus and cells in mice was observed by transmission electron microscope. The samples were fixed with glutaraldehyde (AWI0097; Abiowell, China) and osmic acid (AWI0136, Abiowell, China), dehydrated with acetone gradient, infiltrated with Epon‐812 resin (GS02659; EMCN, China), embedded, and cut into ultrathin sections. The sections were stained with uranium acetate (GZ02625; EMCN, China) and lead citrate (GA10701‐1; EMCN, China), and then observed and imaged by transmission electron microscope (JEM‐1400FLASH; JEOL, Japan), with emphasis on analyzing the morphology and structure of mitochondria. Morphometric quantification of mitochondrial area, perimeter, Feret diameter, aspect ratio, cristae number, and cristae surface area was performed using ImageJ software (Lam et al. 2021; Neikirk, Lopez, et al. 2023; Neikirk, Vue, et al. 2023).

### 2.11 Cell Counting Kit‐8 (CCK8) Assay

Cells were inoculated in a 96‐well plate at the rate of 5 × 10^3^ cells/well and cultured overnight. After the treatment, the medium was replaced with 100 μL fresh medium containing 10% CCK8 reagent (NU679; Dojindo, Japan). After incubation at 37°C for 4 h, the absorbance at 450 nm was measured by an enzyme‐labeled instrument. All experiments were repeated three times.

### 2.12 ATP Content Measurement

According to the instructions of the ATP Assay Kit (A095‐1‐1; NJCB, China), cell samples were homogenized in double‐distilled water, heated in a boiling water bath for 10 min, and centrifuged to collect the supernatant. The supernatant was incubated with substrate solutions I, II, and promoter at 37°C for 30 min. After adding precipitant and centrifugation, the supernatant was reacted with chromogenic agent, and the absorbance was measured at 636 nm. ATP concentration was calculated based on the standard curve and expressed as μmol/g prot.

### 2.13 Flow Cytometry

Apoptosis was detected using the Annexin V‐APC (KGA1030; Meilunbio, China)/PI (MB2920; Meilunbio, China) assay kit. Collected cells were washed with PBS, resuspended in Binding Buffer, Annexin V‐APC and PI dyes were added in turn, and incubated in the dark for 10 min at room temperature. Analysis was performed within 1 h using flow cytometry.

Mitochondrial membrane potential was measured by JC‐1 detection kit (C2006; Beyotime, China). Cells were incubated in JC‐1 working solution at 37°C for 20 min in the dark, washed twice with precooled JC‐1 staining buffer (1 times), resuspended, and analyzed by flow cytometry.

### 2.14 MitoSOX Red Fluorescent Staining

Intracellular superoxide levels were measured using the MitoSOX Red fluorescent probe (HY‐D1055; MCE, USA). Cells were treated with the MitoSOX Red working solution at 37°C in the dark for 20 min. Following a wash with serum‐free medium, the nuclei were counterstained with Hoechst dye. Subsequently, images were viewed and captured using a fluorescence microscope.

### 2.15 RNA Immunoprecipitation PCR (RIP‐qPCR)

Cell lysates were prepared using RIP lysis buffer containing protease inhibitors and RNase, and the lysate was collected. Immunomagnetic beads were generated by incubating specific antibodies m6A antibody (68055‐1‐Ig; Proteintech, China) and HNRNPC antibody (11760‐1‐AP; Proteintech, China), or normal IgG control with protein A/G magnetic beads. Subsequently, the lysate was incubated with the antibody‐magnetic bead complex overnight at 4°C for immunoprecipitation. After washing the magnetic beads multiple times with RIP wash buffer to remove nonspecific binding, proteinase K buffer was added to dissociate the complex. RNA extraction was performed using the TRIZOL (15596026; Thermo, USA)—chloroform method. The extracted RNA was then reverse transcribed into cDNA, followed by PCR analysis using the SYBR Green method. Primers were designed for the target mRNA (YME1L1), and the extent of RNA‐protein binding was evaluated by analyzing *C*_t_ values across the immunoprecipitation group, Input group, and IgG control group to calculate the enrichment fold.

To determine whether the binding between HNRNPC and YME1L1 mRNA is dependent on m6A modification, potential m6A modification sites on YME1L1 mRNA were first predicted using the deepSRAMP online tool (https://www.cuilab.cn/deepsramp/), and three sites with scores > 0.8 were selected for site‐directed mutagenesis (adenine A mutated to thymine T). Wild‐type (WT) or m6A site‐mutated (Mut) YME1L1 transcripts were transfected into HT22 cells. After 48 h, the RIP‐qPCR method described above was used to detect the enrichment level of YME1L1 mRNA by HNRNPC.

### 2.16 RNA Pull Down

Firstly, the biotin‐labeled probe targeting the YME1L1 junction was combined with streptavidin magnetic beads at room temperature for 30 min. The probe‐coated magnetic beads were washed and then treated with total cell protein extract at 4°C for 60 min. The beads were then thoroughly washed with buffer to remove any unbound proteins. Finally, elution buffer was added and incubated at 37°C for 30 min to elute the binding protein complex. Sample buffer (AWB0055; Abiowell, China) was added and boiled to denature the sample. Then the elution products were separated by SDS‐PAGE, and the expression of target proteins (IGF2BP2 and HNRNPC) was detected by WB to analyze the RNA‐protein interaction. In this assay, FL represents the biotin‐labeled full‐length YME1L1 transcript probe, NC represents the negative control probe (used to exclude nonspecific binding), and Input represents the positive control total protein. All experiments were independently repeated six times.

### 2.17 Dual Luciferase Reporter Assay

The wild‐type or mutant m6A site‐containing sequences of YME1L1 were cloned downstream of the pmirGLO reporter vector. The reporter plasmids were co‐transfected into HT22 cells with sh‐HNRNPC or sh‐NC. After 48 h of transfection, firefly luciferase and Renilla luciferase activities were measured using the Dual Luciferase Reporter Assay Kit, and relative luciferase activity was calculated with Renilla luciferase as an internal control.

### 2.18 Quantitative Analysis of Succinylated Protein Modifications

Data Independent Collection (DIA) was used for succinylated protein proteomic analysis of mouse cell samples. Firstly, the total protein was extracted by SDT lysis buffer, and then BCA quantification and SDS‐PAGE quality control were carried out. Subsequently, protein undergoes reduction, alkylation, and trypsin digestion. The succinylated peptides in peptides were enriched by PTMScan succinyl‐lysine motif kit (#13764; Cell Signaling Technology, USA). The enriched peptides were separated by Vanquish Neo UHPLC system (Thermo Scientific, USA) and analyzed by DIA on Orbitrap Astral mass spectrometer (Thermo Scientific, USA). The original data were processed by Spectronaut software (version 19; Biognosys AG, CH), which was used to search UniProt mouse protein database, and succinylation (K) was designated as a variable modification. The analysis finally realized the identification and quantification of succinylated protein, peptides, and sites.

### 2.19 Immunofluorescence Staining

Primary hippocampal neurons grown on coverslips were fixed with 4% paraformaldehyde, permeabilized with 0.3% Triton X‐100, and blocked with 5% BSA. Cells were sequentially incubated with anti‐SIRT5 (1:400, Proteintech, USA) and anti‐HNRNPC (1:400; Proteintech, USA) overnight at 4°C, followed by HRP‐conjugated secondary antibodies and TSA‐520/620 fluorescence reaction (AWI0693a; Abiowell, China). Nuclei were counterstained with DAPI, and images were captured using a fluorescence microscope.

### 2.20 Co‐Immunoprecipitation (Co‐IP)

In order to extract total tissue protein, protease inhibitor (AWH0645; Abiowell, China) was added to IP lysis buffer (AWB0144; Abiowell, China). Then the protein lysate was divided into three parts: one part was treated with HNRNPCs specific antibody, the other part was treated with homologous normal rabbit IgG as negative control, and the other part was kept as input total protein. In order to capture the antigen–antibody complex, the pretreated protein A/G agarose beads were added after rotating incubation at 4°C overnight. Incubation was continued for 2 h. Thereafter, agarose beads were collected by centrifugation and thoroughly washed four times with IP lysis buffer to remove any nonspecific binding. In the last step, the loading buffer should be added and the sample should be boiled to denature it. The analysis involves WB to detect the succinylation and ubiquitination of HNRNPC, and its protein interaction. Mouse monoclonal antibodies against succinyllysine tag (PTM‐419) were purchased from PTM BIO, and against His tag (66005‐1‐Ig), HA (51064‐2‐AP) and ubiquitin (10201‐2‐AP) from Proteintech.

### 2.21 Actinomycin D (ActD) and Cycloheximide (CHX) Assay

Before PCR analysis, cells were treated with actinomycin D (0.5 μg/mL) for 0, 3, 6, 12 and 24 h to determine the stability of mRNA. For protein stability assessment, cells were exposed to cycloheximide (20 μ g/mL) for 0, 3, 6, 9, 12 and 24 h, and then WB analysis was performed.

### 2.22 Statistical Analysis

Statistical analysis was carried out by using GraphPad Prism 8.0 software. The data are expressed as the average standard deviation. The comparison between the two groups uses students' *t*‐test, while the comparison between multiple groups uses one‐way analysis of variance (ANOVA) and then uses Tukey's back testing. The difference is considered to be statistically significant when *p* < 0.05.

## 3 Results

### 3.1 YME1L1 Is Highly Expressed in AD Models and Promotes AD Progression

**FIGURE 1:**
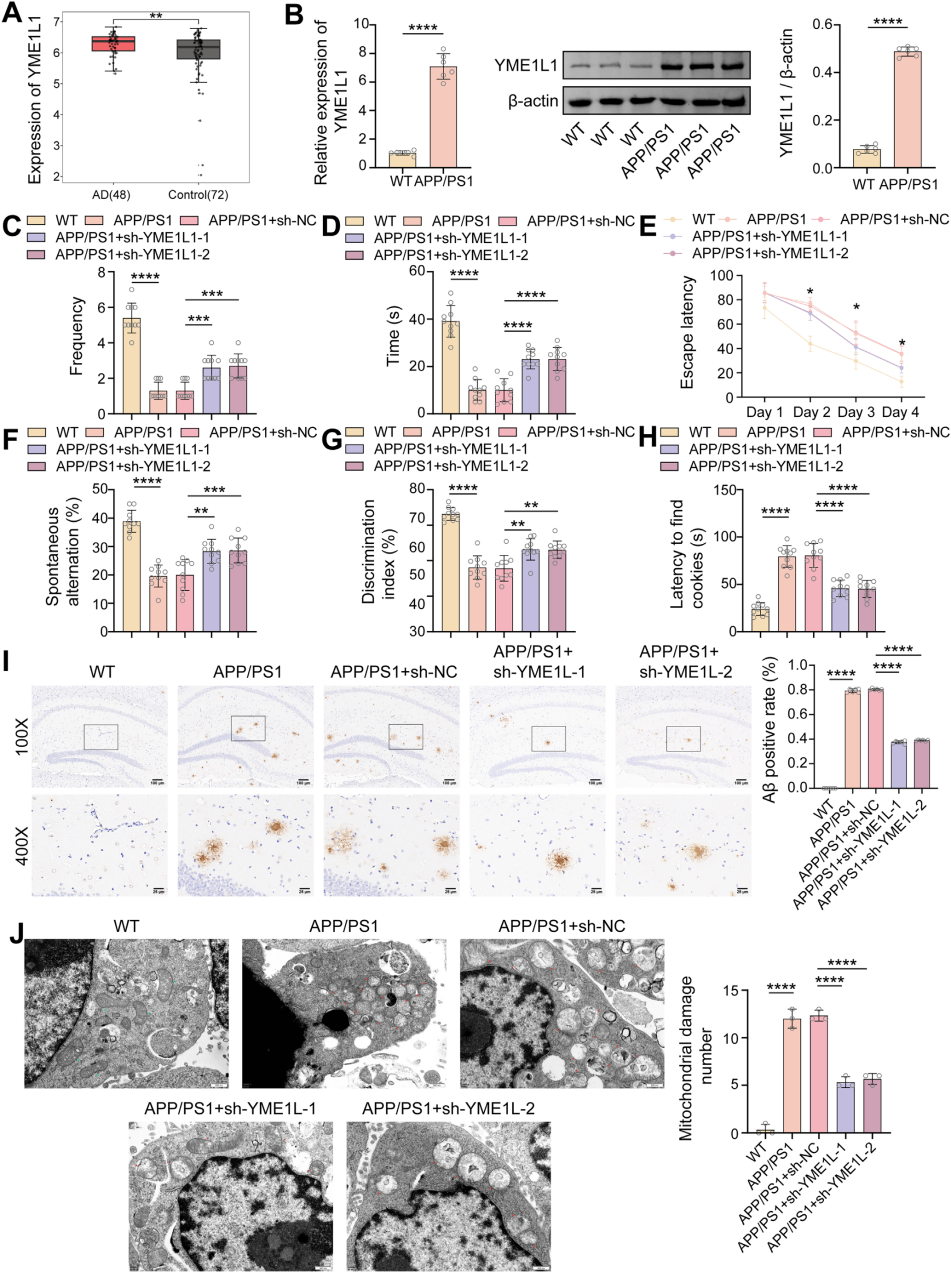
YME1L1 is highly expressed in AD models and promotes AD progression. (A) YME1L1 expression levels in the brain of AD patients based on the GSE48350 dataset. (B) PCR and WB were used to detect the expression of YME1L1 in the hippocampus. (C) Comparison of the frequency of water maze crossing the platform between groups. *n* = 10. (D) The total time spent by mice in the quadrant where the platform was located. *n* = 10. (E) Escape latency to the platform in the Morris water maze during the 4 days before training. *n* = 10. (F) Y‐maze test. *n* = 10. (G) Object recognition test. *n* = 10. (H) Olfactory test. *n* = 10. (I) Aβ immunohistochemical staining of hippocampal tissue. (J) TEM was used to analyze the mitochondrial damage in the hippocampus (The green arrow points to normal mitochondria, and the red arrow points to damaged mitochondria). *n* = 6. **p < 0.01; ***p < 0.001; ****p < 0.0001.

Recent studies indicate that mitochondrial dysfunction plays a pivotal role in the early stages of AD (Spina et al. 2025). The mitochondrial protease YME1L1, as a key protein localized to the inner mitochondrial membrane, has been reported to participate in maintaining mitochondrial functional homeostasis (Xin et al. 2024). Our bioinformatics analysis of publicly available datasets revealed that YME1L1 expression is also significantly elevated in the brain tissue of AD patients (Figure 1A). In order to study the role of YME1L1 in AD, we used APP/PS1 transgenic mice to establish an AD model, and compared it with WT mice. The results showed that compared with the WT group, the protein and mRNA expression levels of YME1L1 were significantly upregulated in the hippocampus of APP/PS1 mice (Figure 1B), which indicated that this protease might play a crucial role in the pathological process of AD. In order to further verify the effect of YME1L1 on the behavior and pathological manifestations of AD, we injected YME1L1 knockdown lentivirus (sh‐YME1L1‐1/2) to inhibit the expression of YME1L1 in the brain of mice. PCR and WB analysis confirmed that both YME1L1 protein and mRNA expression were reduced in the APP/PS1+sh‐YME1L1‐1/2 group, indicating successful lentiviral vector delivery (Figure S1A).

Water maze results showed that compared to the WT group, APP/PS1 mice exhibited reduced platform crossings and target quadrant dwell time. Compared to the APP/PS1+sh‐NC group, the APP/PS1+sh‐YME1L1 group exhibited increased platform crossings and target quadrant dwell time, indicating significant cognitive and memory improvement following YME1L1 knockdown (Figure 1C,D). Escape latency test results showed that compared with the APP/PS1+sh‐NC group, mice in the APP/PS1+sh‐YME1L1 group exhibited shorter escape latencies and superior learning ability (Figure 1E). Y maze experiment shows that the spontaneous alternation rate of the APP/PS1 group is significantly lower than that of the WT group, and knocking down YME1L1 can significantly improve the spontaneous alternation rate (Figure 1F). The ability of the APP/PS1 group to recognize new objects decreased significantly compared with the WT group in the object recognition test, while the ability of sh‐YME1L1‐treated mice to recognize new objects increased significantly (Figure 1G). YME1L1 knockdown significantly enhanced olfactory function in mice, as indicated by olfactory tests (Figure 1H).

IHC staining revealed more severe Aβ deposition in the APP/PS1 group than in the WT group, and YME1L1 knockdown significantly reduced Aβ deposition (Figure 1I). TEM analysis revealed intact mitochondrial architecture in the WT group, severe mitochondrial damage in the APP/PS1 group, and a significant reduction in damaged mitochondria following YME1L1 knockdown (Figure 1J). Morphometric analysis showed that compared to the APP/PS1 group, the YME1L1 knockdown group exhibited increased mitochondrial area, perimeter, Feret diameter, and aspect ratio, as well as increased cristae number and cristae surface area (Figure S2A). The results above indicate that in the AD model, YME1L1 expression is upregulated and promotes neuronal injury and cognitive impairment by disrupting mitochondrial function, suggesting its potential involvement in the pathogenesis of AD.

### 3.2 YME1L1 Promotes Neuronal Mitochondrial Damage After AD

To further elucidate the potential role of YME1L1 in mitochondrial dysfunction associated with AD, we established an AD model using Aβ1‐42‐induced primary hippocampal neuronal cells. First, we screened for effective YME1L1 siRNA knockdown fragments. We separately transfected sh‐YME1L1‐1 and sh‐YME1L1‐2 into primary hippocampal neuronal cells. PCR and WB results showed that, compared to the sh‐NC group, both sh‐YME1L1‐1 and sh‐YME1L1‐2 treatments effectively reduced YME1L1 mRNA and protein expression levels (Figure S3A). The results showed that compared to the sh‐NC group, the sh‐YME1L1‐1 and sh‐YME1L1‐2 groups exhibited slightly decreased cell viability, ATP levels, and mitochondrial membrane potential, as well as slightly increased apoptosis rate and MitoSOX activity. TEM and morphometric quantification of mitochondrial ultrastructure revealed a slight increase in the number of damaged mitochondria, along with mild decreases in mitochondrial area, perimeter, Feret diameter, aspect ratio, cristae number, and cristae surface area (Figure S3B–G).

**FIGURE 2:**
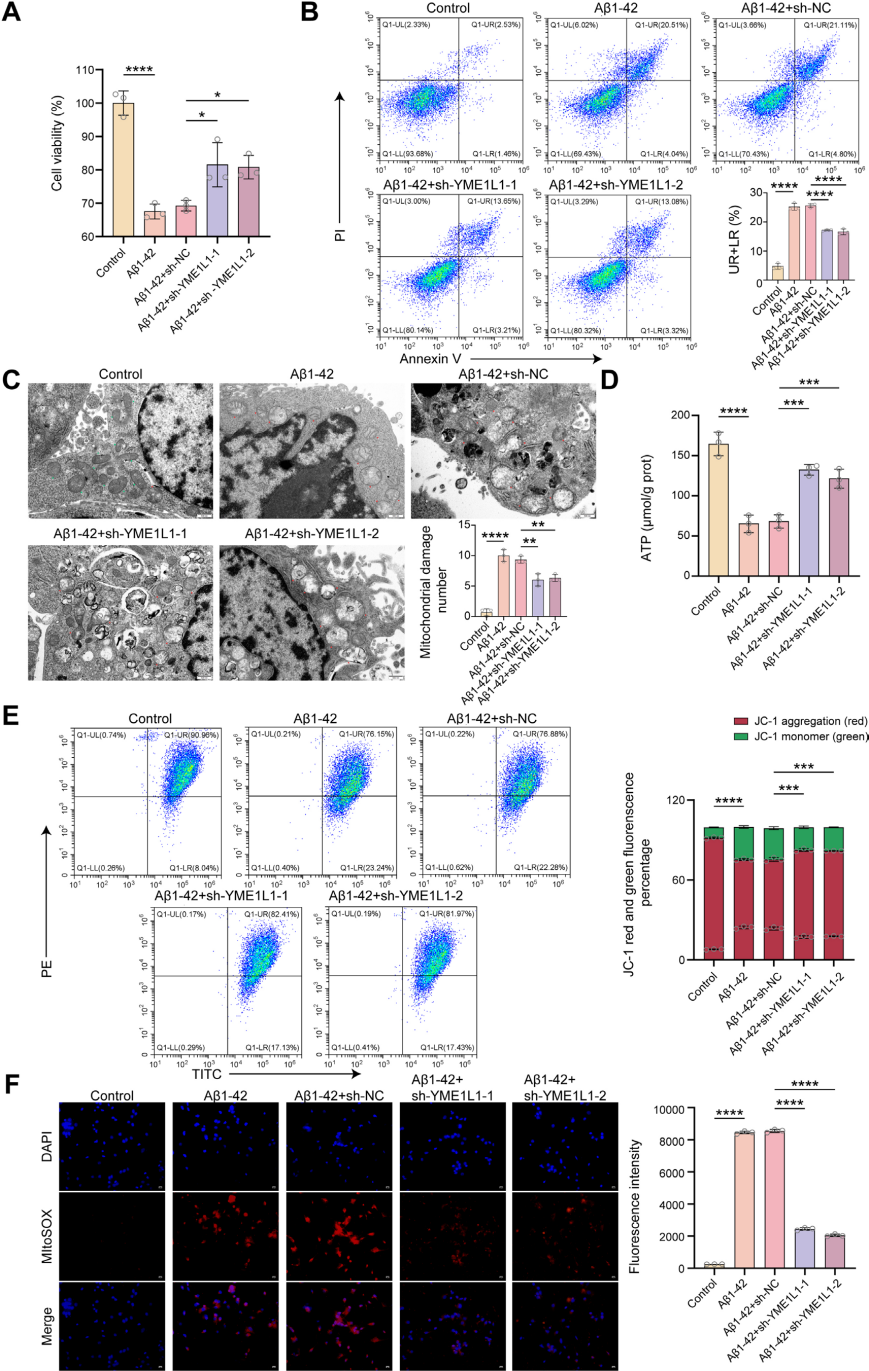
YME1L1 promotes neuronal mitochondrial damage after AD. (A) CCK8 assay for cell viability. (B) Flow cytometry for apoptosis detection. (C) TEM for mitochondrial assessment (The green arrow indicates normal mitochondria, and the red arrow indicates damaged mitochondria). (D) ATP level measurement. (E) JC‐1 staining for cellular mitochondrial membrane potential measurement. (F) MitoSOX fluorescence staining for cellular mitochondrial ROS analysis. *n* = 3. *p < 0.05; **p < 0.01; ***p < 0.001; ****p < 0.0001.

Subsequently, under Aβ‐treated conditions, CCK8 analysis revealed that Aβ1‐42 treatment significantly reduced neuronal viability compared to the control group. Knockdown of YME1L1 markedly attenuated Aβ1‐42‐induced cytotoxicity compared to Aβ1‐42+sh‐NC, resulting in substantial recovery of cell viability (Figure 2A). Aβ1‐42 markedly promoted neuronal apoptosis, which was effectively reversed by YME1L1 knockdown (Figure 2B). TEM analysis of mitochondrial structure revealed that mitochondria in control neurons maintained normal tubular morphology with clearly visible cristae, whereas mitochondria in Aβ1‐42‐treated neurons exhibited marked morphological abnormalities, as evidenced by significantly decreased mitochondrial area, perimeter, Feret diameter, aspect ratio, cristae number, and cristae surface area. Notably, YME1L1 knockdown significantly reversed these alterations (Figure 2C and Figure S4A). At the functional level, compared with the control group, Aβ1‐42 treatment resulted in a significant reduction in neuronal ATP production, whereas YME1L1 knockdown markedly restored ATP levels (Figure 2D). JC‐1 staining revealed that Aβ1‐42 treatment markedly decreased mitochondrial membrane potential (MMP), whereas YME1L1 knockdown effectively maintained MMP stability (Figure 2E). Furthermore, MitoSOX fluorescence staining demonstrated that Aβ1‐42‐induced mitochondrial ROS overproduction was significantly suppressed following YME1L1 knockdown (Figure 2F). These findings indicate that YME1L1 knockdown markedly improves mitochondrial function following AD, alleviates oxidative stress, and reduces apoptosis, suggesting that YME1L1 plays a crucial role in promoting Aβ1‐42‐induced neuronal mitochondrial damage.

### 3.3 YME1L1 Induces Neuronal Mitochondrial Damage and Neuronal Death Following AD Through OPA1 Hydrolysis

**FIGURE 3:**
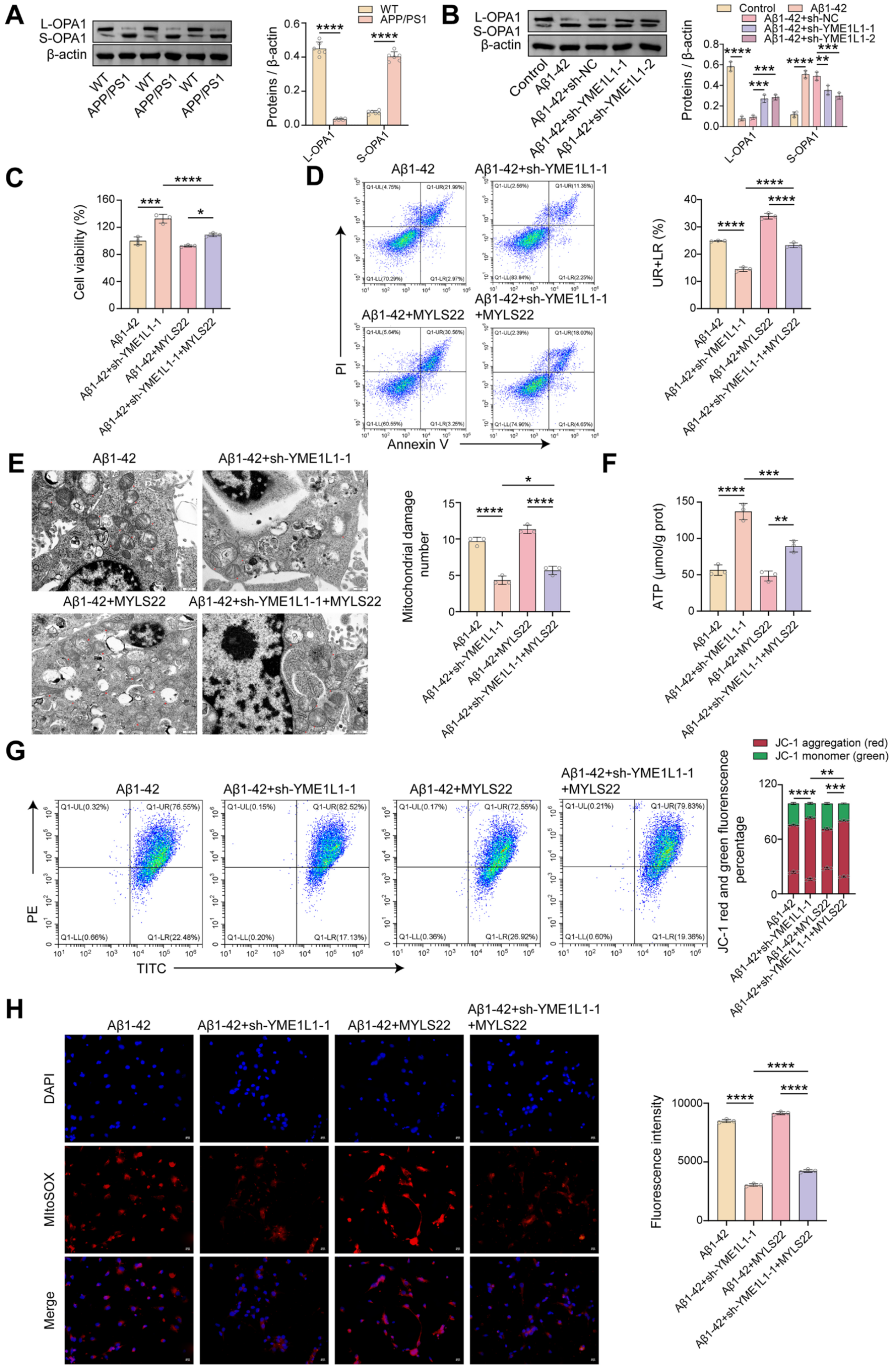
YME1L1 induces neuronal mitochondrial damage and neuronal death following AD through OPA1 hydrolysis. (A) WB detection of OPA1 expression in hippocampal tissue (*n* = 6). (B) WB detection of OPA1 expression. (C) CCK8 assay for cell viability. (D) Flow cytometry for apoptosis detection. (E) TEM for mitochondrial detection (The red arrow indicates damaged mitochondria). (F) ATP level detection. (G) JC‐1 staining for measuring mitochondrial membrane potential. (H) MitoSOX fluorescence staining for analyzing mitochondrial ROS. *n* = 3. *p < 0.05; **p < 0.01; ***p < 0.001; ****p < 0.0001.

OPA1 is a key regulatory protein in the mitochondrial fusion process, comprising two subtypes: S‐OPA1 and L‐OPA1. The function of OPA1 is regulated by the hydrolysis and modulation of the mitochondrial protease YME1L1 (Grel et al. 2023). Under steady state conditions, L‐OPA1 and S‐OPA1 coexist; in damaged mitochondria, the majority of L‐OPA1 is converted to S‐OPA1 and loses its fusion activity (Ban et al. 2017). Therefore, the dynamic equilibrium between L‐OPA1 and S‐OPA1 is crucial for maintaining mitochondrial morphology and function (Wai et al. 2015). To investigate whether YME1L1 participates in mitochondrial dysfunction and neuronal death during AD by regulating OPA1 hydrolysis, we first examined OPA1 expression in hippocampal tissue from APP/PS1 mice. Compared to WT mice, APP/PS1 mice exhibited significantly elevated S‐OPA1 expression and markedly reduced L‐OPA1 expression in hippocampal tissue (Figure 3A), indicating a disrupted L‐OPA1 to S‐OPA1 ratio in the AD model. To exclude the possibility that other mitochondrial stress proteases, such as OMA1, participate in OPA1 hydrolysis, we examined OMA1 expression levels in the hippocampus of APP/PS1 mice. The results showed that compared with the WT group, OMA1 protein expression was mildly upregulated in the hippocampus of APP/PS1 mice (Figure S5A), whereas YME1L1 expression was significantly upregulated (Figure 1B).

In an Aβ1‐42‐induced primary AD hippocampal neuronal cell model, we further validated the regulatory role of YME1L1 on OPA1. WB analysis revealed that S‐OPA1 expression significantly increased while L‐OPA1 expression decreased following Aβ1‐42 treatment, and YME1L1 knockdown effectively reversed this process (Figure 3B). To clarify the role of OPA1 in YME1L1‐mediated neuroprotection and mitochondrial function maintenance, cells were treated with MYLS22, a specific inhibitor of L‐OPA1. Knockdown of YME1L1 markedly improved Aβ1‐42‐induced neuronal viability decline, whereas MYLS22 reversed the protective effect of YME1L1 knockdown (Figure 3C). Knockdown of YME1L1 significantly suppressed Aβ1‐42‐induced neuronal apoptosis, whereas MYLS22 treatment attenuated this antiapoptotic effect (Figure 3D). TEM observation revealed that knocking down YME1L1 significantly alleviated Aβ1‐42‐induced mitochondrial damage, as evidenced by the restoration of mitochondrial area, perimeter, Feret diameter, aspect ratio, cristae number, and cristae surface area, while MYLS22 treatment markedly attenuated the effects of sh‐YME1L1 (Figure 3E and Figure S4B). Functionally, YME1L1 knockdown significantly increased ATP production (Figure 3F), maintained mitochondrial membrane potential stability (Figure 3G), and suppressed excessive mitochondrial ROS generation (Figure 3H). However, MYLS22 treatment partially reversed these beneficial effects.

To further validate that mitochondrial dysfunction and neuronal death occur specifically through OPA1 processing downstream of YME1L1, we performed S‐OPA1 overexpression rescue experiments in primary hippocampal neurons. The results showed that S‐OPA1 overexpression further exacerbated Aβ1‐42‐induced reduction in cell viability (Figure S5B) and increase in apoptosis (Figure S5C). Notably, S‐OPA1 overexpression in the context of YME1L1 knockdown significantly reversed the protective effects of sh‐YME1L1 (Figure S4B,C). TEM observation revealed that S‐OPA1 overexpression in the sh‐YME1L1 background partially reversed the mitochondrial protective effects of YME1L1 knockdown, as evidenced by reduced mitochondrial area, perimeter, Feret diameter, aspect ratio, cristae number, and cristae surface area (Figure S5D). Functional assays further confirmed that S‐OPA1 overexpression in the sh‐YME1L1 background partially reversed the sh‐YME1L1‐mediated protective effects on ATP levels, mitochondrial membrane potential, and ROS production (Figure S3E–G). These findings indicate that YME1L1 disrupts the physiological balance between L‐OPA1 and S‐OPA1 by promoting OPA1 hydrolysis. This disruption leads to decreased L‐OPA1 levels and increased S‐OPA1 levels, thereby causing mitochondrial dysfunction and neuronal death, with S‐OPA1 serving as a key downstream effector molecule of YME1L1.

### 3.4 The High Expression of YME1L1 in AD Is Regulated by HNRNPC

**FIGURE 4:**
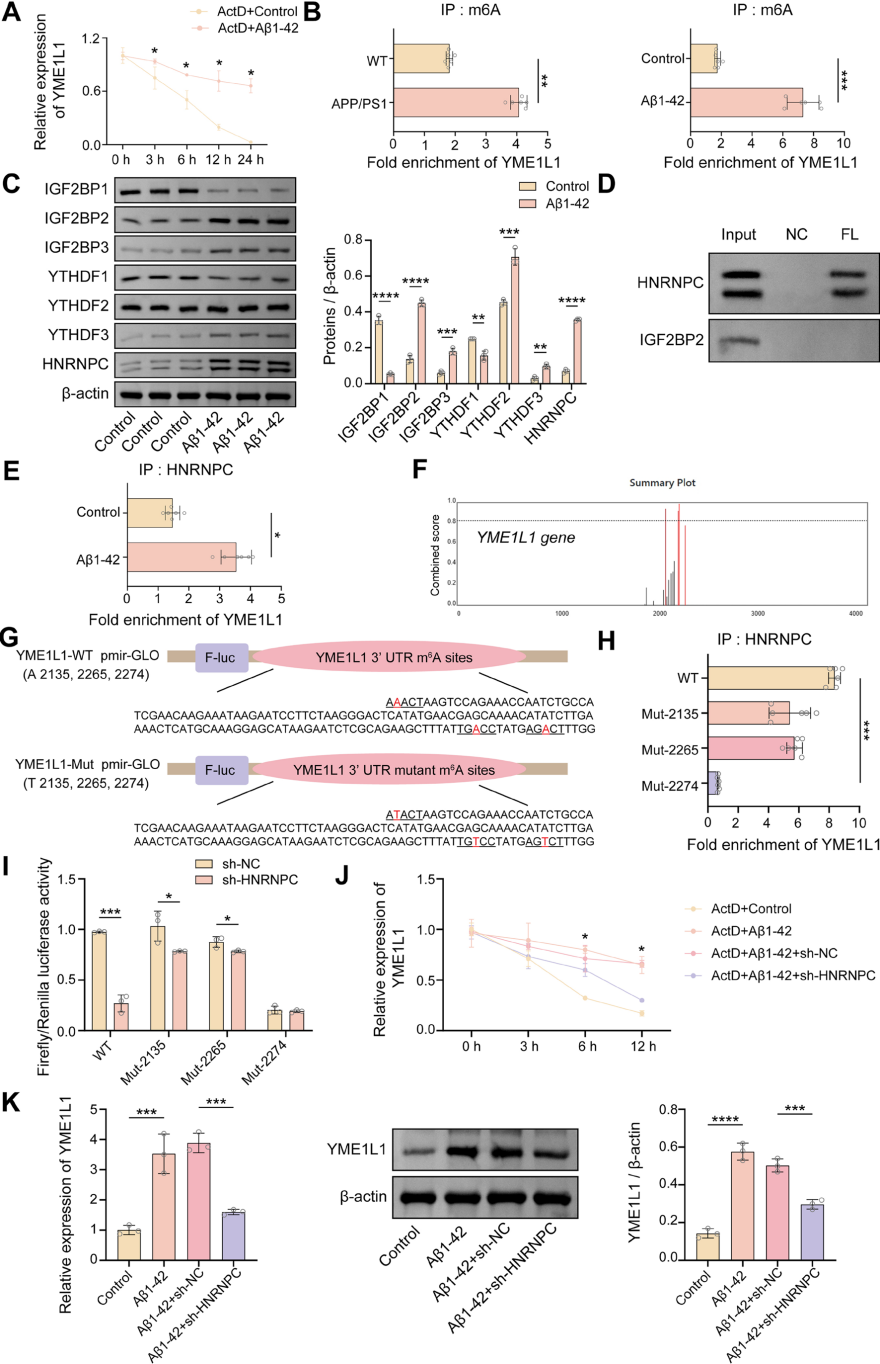
The high expression of YME1L1 in AD is regulated by HNRNPC. (A) ActD was used to determine the mRNA stability of YME1L1. (B) m6A enriched YME1L1 in neuronal cells through m6A‐RIP‐qPCR. *n* = 6. (C) WB detected the expression of IGF2BP1, IGF2BP2, IGF2BP3, YTHDF1, YTHDF2, YTHDF3, and HNRNPC. (D) RNA pull‐down screened the m6A reader proteins IGF2BP2 and HNRNPC that bind to YME1L1. *n* = 6. (E) RIP–qPCR analysis showed that YME1L1 transcripts were immunoprecipitated by HNRNPC. *n* = 6. (F) Prediction of m6A modification site of YME1L1 by deepSRAMP online tool. (G) The diagram shows the relative positions of three predicted m6A modification sites (sites 2274, 2265, and 2135) within the YME1L1 mRNA transcript. Red asterisks indicate the mutation sites generated in this study. (H) RIP‐qPCR analysis of the effect of m6A site mutation on the binding between HNRNPC and YME1L1 mRNA. *n* = 6. (I) The m6A dependence of HNRNPC‐YME1L1 mRNA binding was verified by Dual luciferase reporter assay. (J) Primary neuronal cells were transfected with sh‐HNRNPC or sh‐NC and treated with ActD for the indicated time in the absence or presence of Aβ1‐42. Cells were collected for PCR analysis of YME1L1. (K) PCR and WB were performed to detect the expression of YME1L1. *n* = 3. *p < 0.05; **p < 0.01; ***p < 0.001; ****p < 0.0001.

Given the overexpression of YME1L1 in AD and its role in disease progression, we investigated its potential mechanisms. Our ActD experiment demonstrated that treatment with Aβ1‐42 significantly reduced the degradation rate of YME1L1 mRNA (Figure 4A), indicating a potential association between increased mRNA stability and YME1L1 expression in AD. The crucial function of m6A in controlling mRNA stability has been shown in numerous investigations (Wang et al. 2023; Zhang et al. 2024). Hippocampal tissue from APP/PS1 mice and Aβ1‐42‐treated neurons had markedly higher m6A modification levels in YME1L1, according to m6A‐RIP‐qPCR results (Figure 4B). According to reports, m6A‐binding proteins, including YTHDF1, YTHDF2, and YTHDF3, as well as members of the insulin‐like growth factor 2‐binding protein (IGF2BP) family (IGF2BP1, 2, and 3), are implicated in recognizing m6A modifications (Shi et al. 2018). Additionally, the m6A reader proteins encompass the RNA‐binding protein HNRNPC (Rong et al. 2024). Following Aβ1‐42 treatment, WB analysis revealed a significant upregulation of m6A reader proteins HNRNPC, IGF2BP2, IGF2BP3, YTHDF2, and YTHDF3 in the AD model, among which IGF2BP2 and HNRNPC were the most upregulated. Conversely, IGF2BP1 and YTHDF1 were downregulated (Figure 4C).

Through RNA pull‐down experiments, we found that the biotin‐labeled full‐length YME1L1 probe (FL) specifically pulled down HNRNPC, whereas no HNRNPC band was detected with the negative control probe (NC). IGF2BP2 showed no significant binding in either the FL or NC group, indicating that HNRNPC directly and specifically binds to YME1L1 mRNA (Figure 4D). Further RIP‐qPCR analysis confirmed that Aβ1‐42 treatment significantly enhanced the binding capacity between HNRNPC and YME1L1 (Figure 4E). Notably, we observed significantly upregulated HNRNPC expression in the AD in vivo and in vitro model (Figure S6A,B) and successfully identified an effective sh‐HNRNPC knockdown fragment (Figure S6C). To determine whether the binding between HNRNPC and YME1L1 mRNA is dependent on m6A modification, we first predicted potential m6A modification sites on YME1L1 mRNA using the deepSRAMP online tool (Figure 4F) and generated mutations at three sites with scores > 0.8 (Figure 4G). RIP‐qPCR results showed that mutation of m6A sites 2274, 2265, or 2135 all reduced the enrichment of YME1L1 mRNA by HNRNPC, with the most pronounced effect observed upon mutation of site 2274 (Figure 4H). Dual luciferase reporter assays further demonstrated that when the sequence containing the wild‐type m6A site was cloned downstream of the reporter gene, HNRNPC knockdown significantly inhibited luciferase activity; however, mutation of the m6A site 2274 abolished this effect, whereas mutation of the sites 2265 or 2135 had no such effect (Figure 4I). These results indicate that the binding of HNRNPC to YME1L1 mRNA is dependent on the m6A modification site 2274 within the YME1L1 transcript.

To validate the role of HNRNPC in regulating YME1L1 mRNA stability, we measured the degradation rate of YME1L1 mRNA under ActD treatment conditions. Experimental results indicate that Aβ1‐42 treatment significantly delayed YME1L1 mRNA degradation, while HNRNPC knockdown partially reversed this stabilizing effect (Figure 4J) and markedly reduced YME1L1 expression levels (Figure 4K). These findings suggest that HNRNPC is involved in the Aβ‐induced upregulation of YME1L1 expression by directly binding to YME1L1 mRNA and enhancing its stability.

### 3.5 Succinylation Promotes the Protein Stability of HNRNPC

**FIGURE 5:**
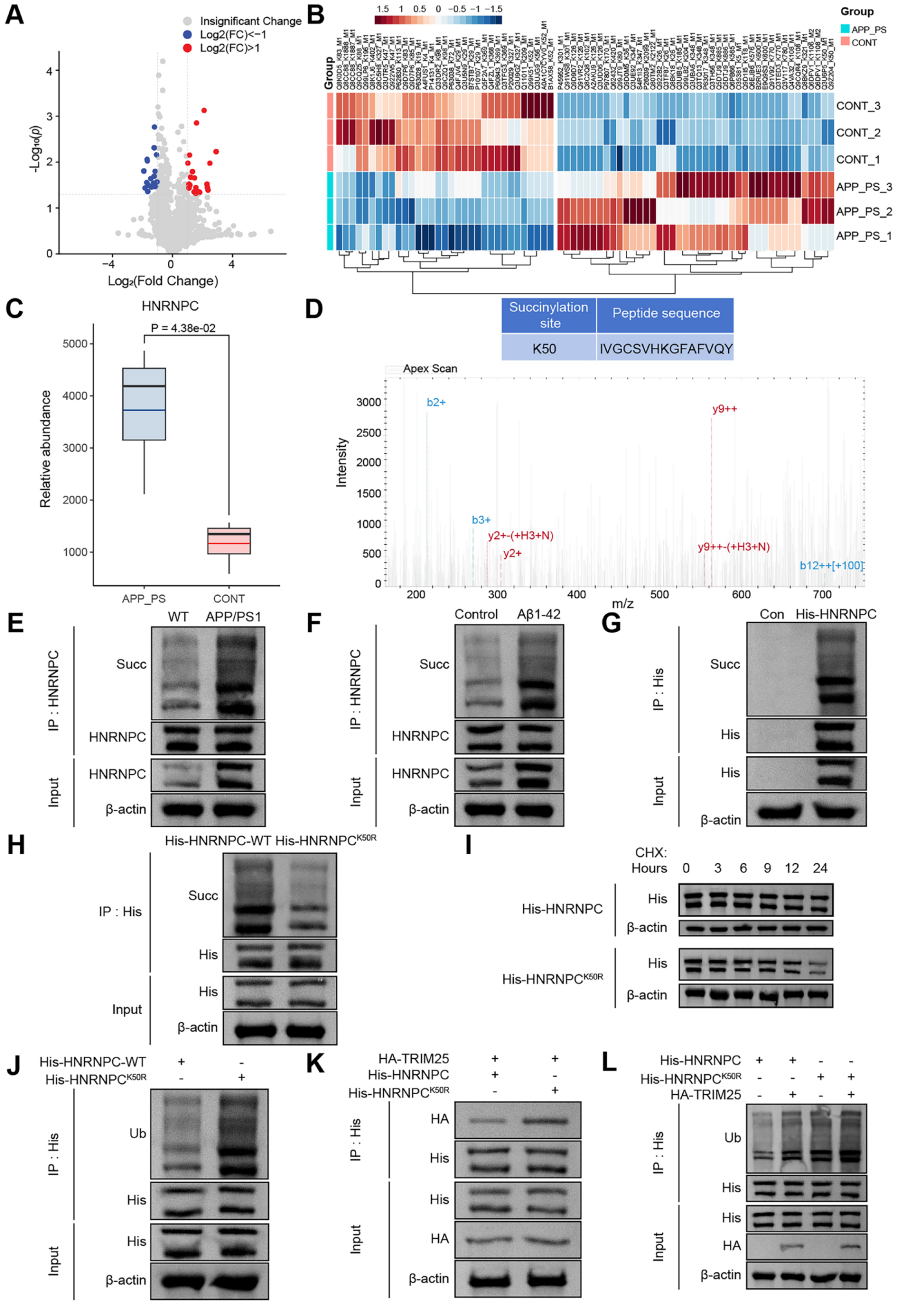
Succinylation promotes the protein stability of HNRNPC. (A) Volcano plot showing differentially succinylated proteins. (B) Heat map showing upregulated succinylated proteins. (C) Proteomics identified lysine succinylation expression of HNRNPC in two groups. (D) Succinylation sequence of HNRNPC at K50 site. (E) Co‐IP detection of HNRNPC succinylation in hippocampus tissue. (F) Co‐IP detection of HNRNPC succinylation. (G) His‐HNRNPC was transfected into HT22 cells, and the succinylation level of HNRNPC was detected by co‐IP. (H) His‐HNRNPC‐WT and His‐HNRNPCK50R were transfected into HT22 cells, and the succinylation level of HNRNPC was detected by co‐IP. (I) His‐HNRNPC‐WT, His‐HNRNPC^K50R^ were transfected into HT22 cells, and the protein degradation rate of HNRNPC was detected by WB. (J) The ubiquitination level of HNRNPC was detected by Co‐IP. (K) Co‐IP was used to detect the binding of TRIM25 and HNRNPC in HT22 cells transfected with HA‐TRIM25 and His‐HNRNPC or His‐HNRNPC^K50R^. (L) Co‐IP was used to detect the ubiquitination of HNRNPC in HT22 cells transfected with His‐HNRNPC or His‐HNRNPC^K50R^ with or without HA‐TRIM25. *n* = 3.

Protein succinylation is a crucial posttranslational modification (Trefely et al. 2020). Our investigation delved into whether succinylation impacts HNRNPC abundance and activity by influencing protein stability. Succinylation proteomics analysis unveiled notable changes in succinylation levels among numerous proteins in Aβ1‐42‐treated HT22 cells (Figure 5A,B). Notably, HNRNPC exhibited significantly elevated succinylation levels following Aβ1‐42 treatment (Figure 5C), with the K50 site identified as the primary succinylation modification site (Figure 5D). Validation experiments in AD models demonstrated markedly elevated HNRNPC succinylation levels in hippocampal tissue from APP/PS1 mice (Figure 5E) and in Aβ‐treated primary neurons (Figure 5F). To further investigate the impact of succinylation on HNRNPC function, we transfected wild‐type (WT‐HNRNPC) and K50R point mutation plasmids (HNRNPC^K50R^) into HT22 cells. Experiments confirmed that the succinylation level of the HNRNPC^K50R^ mutant was significantly lower than that of the wild‐type (Figure 5G,H), indicating that K50 is a critical succinylation site on HNRNPC. CHX chase experiments revealed that the HNRNPC^K50R^ mutant degraded significantly faster than the WT (Figure 5I). Mechanistically, the K50R mutation significantly enhances the ubiquitination level of HNRNPC (Figure 5J), suggesting that succinylation modification may stabilize the HNRNPC protein by inhibiting the ubiquitination pathway. TRIM25, a member of the E3 ubiquitin ligase tripartite motif family, mediates the ubiquitination modification of HNRNPC (Zheng et al. 2024). Co‐IP experiments revealed that TRIM25 more readily bound to the dessuccinylated HNRNPC^K50R^ mutant and increased the ubiquitination of HNRNPC^K50R^ mutant (Figure 5K,L). The above results collectively indicate that succinylation at the K50 site of HNRNPC enhances protein stability by inhibiting TRIM25‐mediated ubiquitin‐dependent degradation, thereby promoting its accumulation in AD.

### 3.6 HNRNPC Succinylation Modifies Neuronal Mitochondrial Damage and Neuronal Death Following AD via YME1L1

**FIGURE 6:**
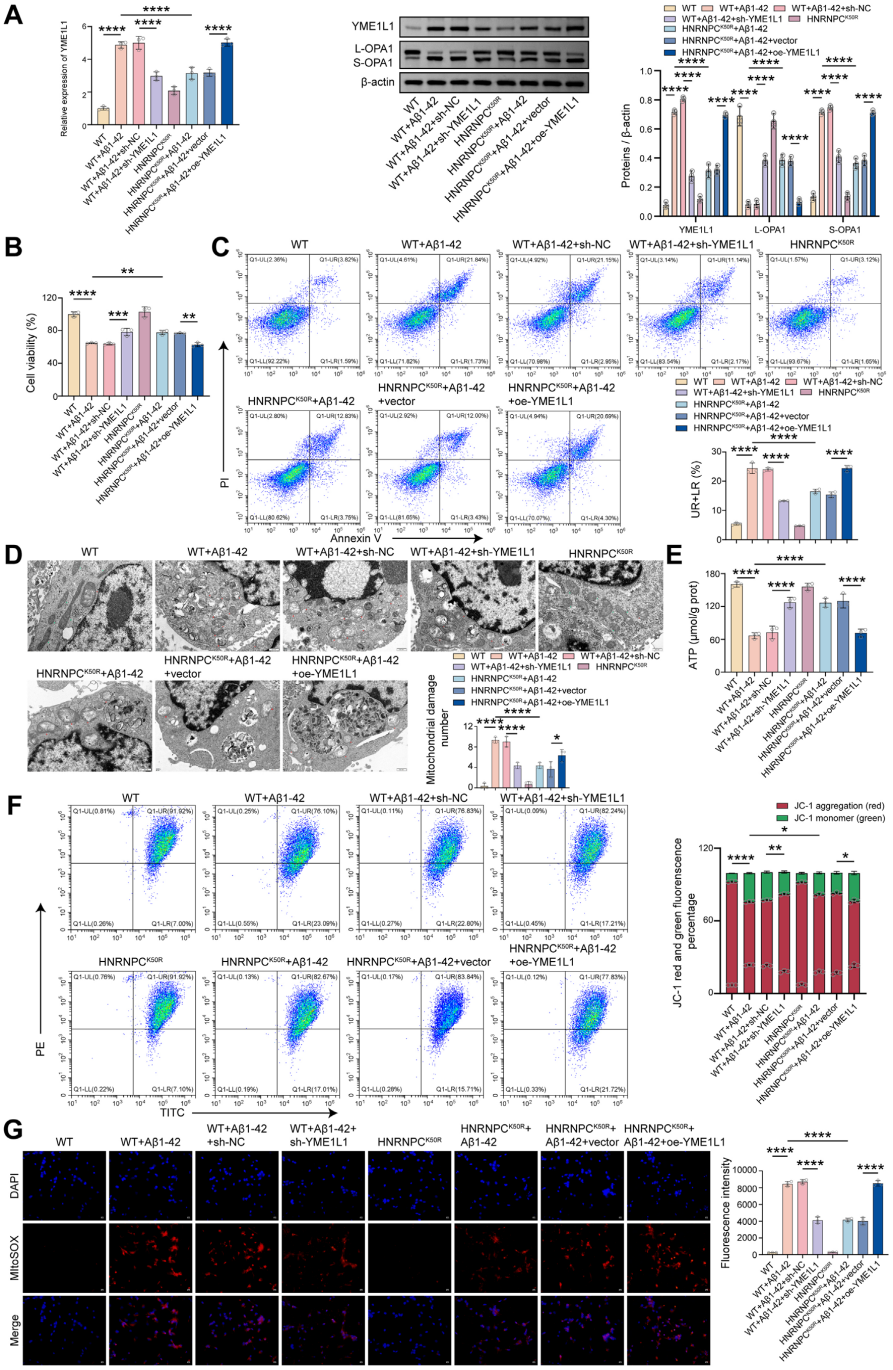
HNRNPC succinylation modifies neuronal mitochondrial damage and neuronal death following AD via YME1L1. (A) PCR and WB detection of YME1L1, WB detection of OPA1. (B) CCK8 analysis of cell activity. (C) Flow cytometry to detect cell apoptosis. (D) TEM detection of mitochondria (The green arrow points to normal mitochondria, and the red arrow points to damaged mitochondria). (E) ATP level detection. (F) JC‐1 staining was used to measure the mitochondrial membrane potential of cells. (G) MitoSOX fluorescent staining analysis of mitochondrial ROS in cells. *n* = 3. *p < 0.05; **p < 0.01; ***p < 0.001; ****p < 0.0001.

To validate whether HNRNPC succinylation mediates neuronal mitochondrial dysfunction and cell death in AD by regulating YME1L1 expression, we separately transfected sh‐YME1L1, oe‐YME1L1, and HNRNPC^K50R^ mutant plasmids into primary neuronal cells and systematically evaluated their effects on mitochondrial function and cell survival. Experimental results demonstrated that Aβ1‐42 treatment significantly upregulated YME1L1 expression and caused the hydrolysis imbalance of OPA1 (decreased L‐OPA1 and increased S‐OPA1), while HNRNPC^K50R^ mutation or YME1L1 knockdown reverses this phenomenon (Figure 6A). Notably, overexpression of YME1L1 in the context of the HNRNPC^K50R^ mutation completely counteracts the effects induced by HNRNPC^K50R^, confirming that YME1L1 is a key downstream effector of HNRNPC succinylation (Figure 6A). Functional assays demonstrated that both the HNRNPC^K50R^ mutant and YME1L1 knockdown significantly improved cell viability (Figure 6B) and reduced apoptosis (Figure 6C), whereas overexpression of YME1L1 abolished the protective effect conferred by HNRNPC^K50R^. TEM observation showed that HNRNPC^K50R^ and YME1L1 knockdown significantly reduced the mitochondrial ultrastructure damage induced by Aβ1‐42, as evidenced by increased mitochondrial area, perimeter, Feret diameter, aspect ratio, cristae number, and cristae surface area. Similarly, overexpression of YME1L1 reversed this beneficial effect (Figure 6D and Figure S7A). In terms of energy metabolism, the HNRNPC^K50R^ and YME1L1 knockdown groups showed significant recovery of ATP level (Figure 6E) and mitochondrial membrane potential (Figure 6F), while the level of mitochondrial ROS decreased significantly (Figure 6G). It is worth noting that the overexpression of YME1L1 counteracts the beneficial effect of HNRNPCK^50R^ and emphasizes the key function of YME1L1 as the key downstream effector of HNRNPC succinylation. The above results indicate that succinylation modification at the K50 site of HNRNPC promotes the upregulation of YME1L1 expression by stabilizing the HNRNPC protein, thereby leading to OPA1 hydrolysis imbalance and mitochondrial dysfunction.

### 3.7 SIRT5 Affects the Desuccinylation of HNRNPC in AD Models

Sirtuins (SIRT) are a class of evolutionarily conserved nicotinamide adenine dinucleotide (NAD)‐dependent deacylases comprising seven members: SIRT1 to SIRT7 (Lagunas‐Rangel 2025). Research indicates that SIRT5, localized to mitochondria, exhibits not only weak deacetylase activity but also potent desuccinylase activity on lysine residues (Liu et al. 2020; Yihan et al. 2021). SIRT7 similarly possesses desuccinylase activity (Yuan et al. 2022). However, the regulatory role of SIRT5 or SIRT7 on HNRNPC in AD remains unclear. Therefore, we subsequently investigated whether SIRT5 or SIRT7 could regulate the succinylation status of HNRNPC in AD and influence its function. We first examined the expression levels of SIRT5 and SIRT7 in the AD model. Results showed that compared to WT mice, both SIRT5 and SIRT7 protein expression was significantly reduced in the hippocampal tissue of APP/PS1 mice (Figure S8A). Downregulation of SIRT5 and SIRT7 was similarly observed in Aβ1‐42‐treated primary neurons (Figure S8B), suggesting impaired desuccinylase activity in the AD pathological environment.

**FIGURE 7:**
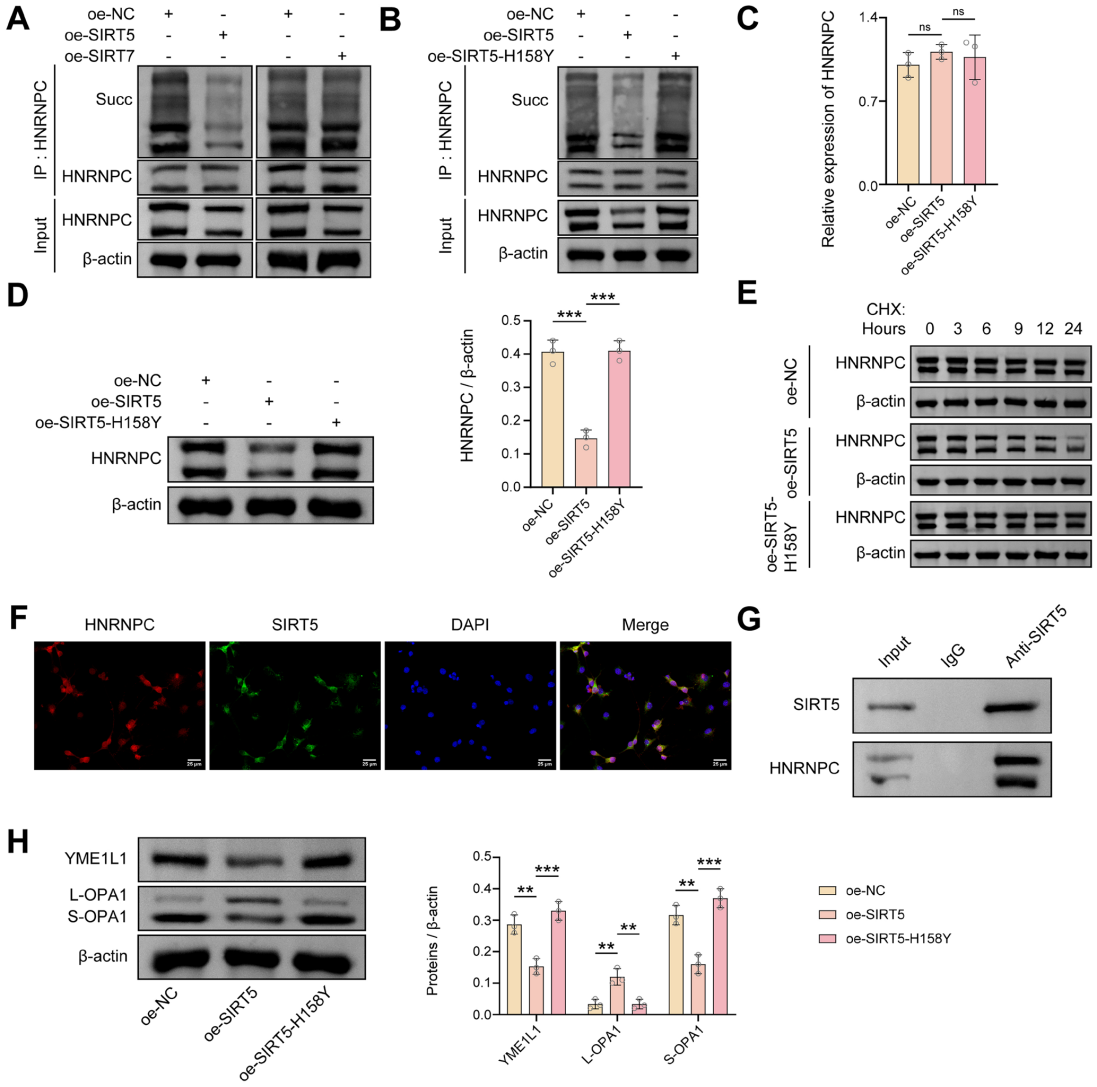
SIRT5 affects the desuccinylation of HNRNPC in AD models. (A, B) Co‐IP detection of HNRNPC succinylation. (C) PCR detection of HNRNPC mRNA level. (D) WB detection of HNRNPC protein level. (E) Cells were treated with CHX (20 μg/mL) for 0, 3, 6, 9, 12, and 24 h. The degradation rate of HNRNPC was detected by WB. (F) Immunofluorescence detection of colocalization between SIRT5 and HNRNPC in neuronal cells. Scale bar = 25 μm. (G) Co‐IP was used to detect the interaction between SIRT5 and HNRNPC. (H) The expression of YME1L1 and OPA1 was detected by WB. *n* = 3. *p < 0.05; **p < 0.01; ***p < 0.001.

In order to determine the SIRT members involved in the desuccinylation of HNRNPC, we overexpressed SIRT5 or SIRT7 in HT22 cells treated with Aβ1‐42, respectively (Figure S8C,D). Co‐IP experiments showed that overexpression of SIRT5 significantly reduced the succinylation level of HNRNPC, while overexpression of SIRT7 had no such effect (Figure 7A), indicating that SIRT5 was the key enzyme to regulate the succinylation of HNRNPC. In order to further verify the enzyme activity dependence of SIRT5, we introduced an enzyme‐deficient mutant SIRT5‐H158Y. The results showed that overexpression of SIRT5 significantly reduced the succinylation of HNRNPC, while the mutant SIRT5‐H158Y had no such effect (Figure 7B), indicating that SIRT5 mediated the succinylation of HNRNPC in an enzyme activity‐dependent manner. In addition, the overexpression of SIRT5 did not affect the mRNA level of HNRNPC, but significantly reduced its protein level (Figure 7C,D) and accelerated its degradation (Figure 7E), but the mutant SIRT5‐H158Y had no significant effect on the mRNA, protein level and degradation rate of HNRNPC. Immunofluorescence staining revealed significant colocalization of SIRT5 and HNRNPC in neuronal cells (Figure 7F), suggesting a potential direct interaction between the two proteins in cells. Co‐IP experiments further confirmed a direct interaction between SIRT5 and HNRNPC (Figure 7G). More importantly, overexpression of SIRT5 downregulated YME1L1 expression and restored the imbalance in OPA1 hydrolysis (increased L‐OPA1 and decreased S‐OPA1) (Figure 7H), whereas the enzyme activity‐deficient mutant SIRT5‐H158Y did not exhibit this effect. Based on the aforementioned findings, SIRT5 overexpression in the AD model leads to increased desuccinylation activity, thereby reducing the succinylation modification of HNRNPC and decreasing its protein stability.

### 3.8 SIRT5 Affects Neuronal Mitochondrial Function by Desuccinylating HNRNPC

**FIGURE 8:**
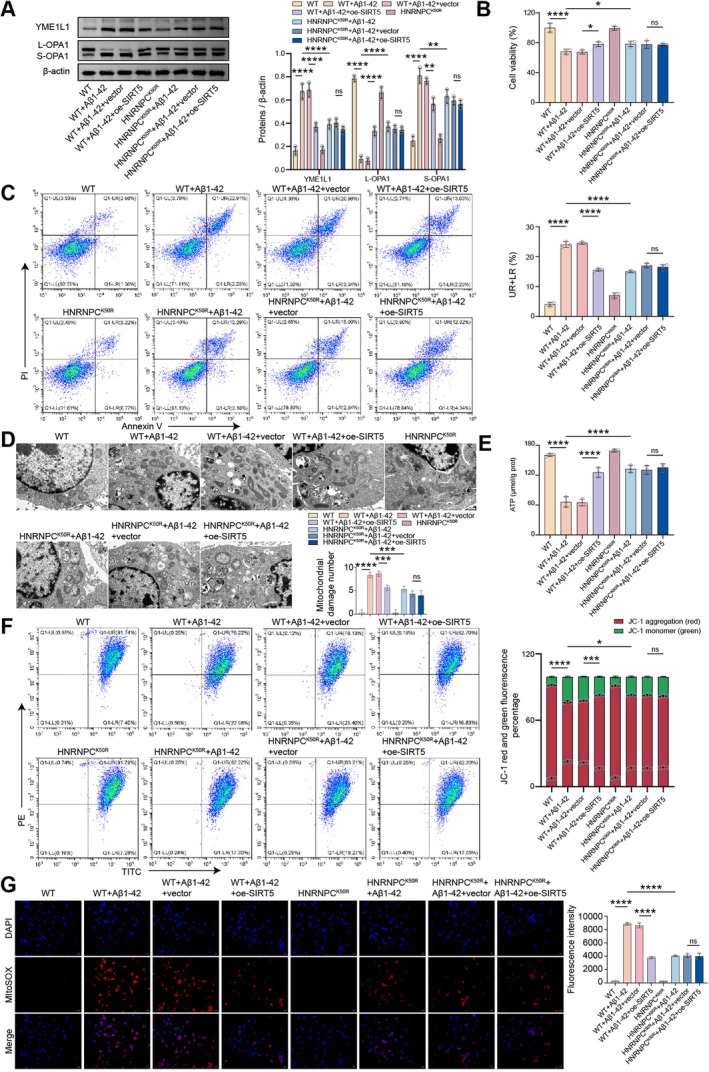
SIRT5 affects neuronal mitochondrial function by desuccinylating HNRNPC. (A) WB detection of YME1L1 and OPA1 expression. (B) CCK8 analysis of cell activity. (C) Flow cytometry detection of cell apoptosis. (D) TEM detection of mitochondrial morphology (The green arrow points to normal mitochondria, and the red arrow points to damaged mitochondria). (E) ATP level detection. (F) JC‐1 staining to measure cell mitochondrial membrane potential. (G) MitoSOX fluorescent staining analysis of mitochondrial ROS in cells. *n* = 3. *p < 0.05; **p < 0.01; ***p < 0.001; ****p < 0.0001; ns stands for no significant difference.

To determine whether SIRT5 influences neuronal mitochondrial function by regulating the succinylation status of HNRNPC, we overexpressed SIRT5 and HNRNPC^K50^ in a primary neuronal cell model damaged by Aβ1‐42. SIRT5 overexpression significantly inhibited the Aβ1‐42‐induced upregulation of YME1L1 expression and the imbalance in OPA1 hydrolysis (decreased L‐OPA1, increased S‐OPA1). Similar ameliorative effects were observed in the HNRNPC^K50R^ group (Figure 8A). Notably, in HNRNPC K50R mutant cells, overexpression of SIRT5 failed to further improve the imbalance in OPA1 hydrolysis, indicating that SIRT5 primarily functions by targeting the HNRNPC K50 site (Figure 8A). Overexpression of either SIRT5 or HNRNPC^K50^ significantly increased cell viability (Figure 8B) and reduced apoptosis (Figure 8C). TEM revealed that SIRT5 overexpression effectively mitigated Aβ1‐42‐induced mitochondrial ultrastructural damage, as evidenced by increased mitochondrial area, perimeter, Feret diameter, aspect ratio, cristae number, and cristae surface area. However, this protective effect did not exist in cells with a mutation of the HNRNPC K50R residue (Figure 8D and Figure S9A), confirming that the HNRNPC K50 site is the key target of SIRT5 function. Additionally, ATP levels (Figure 8E) and mitochondrial membrane potential (Figure 8F) were significantly improved in both the SIRT5‐overexpressing and HNRNPC^K50^ groups, while mitochondrial ROS levels were markedly reduced in these two groups (Figure 8G). Similarly, under the HNRNPC K50R mutation background, SIRT5 overexpression failed to further improve these metrics. These results suggest that SIRT5 enhances HNRNPC degradation by targeting desuccinylation at the K50 site, thereby repressing YME1L1 expression and enhancing mitochondrial function.

### 3.9 SIRT5 Improves Cognition and Reduces Aβ Deposition in AD Mice by Desuccinylating HNRNPC

**FIGURE 9:**
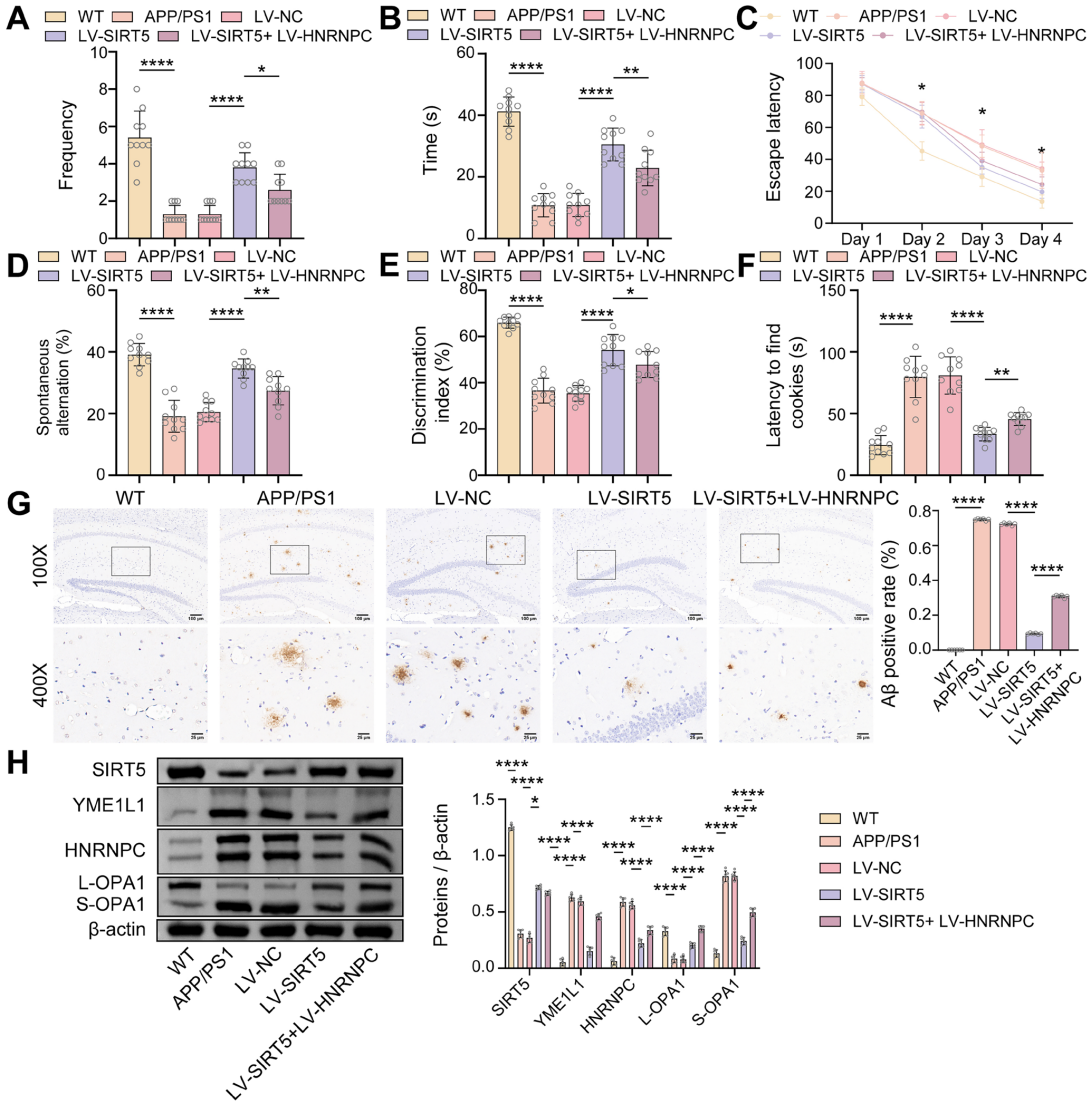
SIRT5 improves cognition and reduces Aβ deposition in AD mice by desuccinylating HNRNPC. (A) Comparison of the frequency of crossing the platform in the water maze between groups. *n* = 10. (B) The total time spent by mice in the quadrant where the platform is located. *n* = 10. (C) Escape latency from the water maze to the platform within 4 days before training. *n* = 10. (D) Y‐maze test. *n* = 10. (E) Object recognition test. *n* = 10. (F) Olfactory test. *n* = 10. (G) Aβ immunohistochemical staining of hippocampal tissue. (H) WB detected the expression of SIRT5, HNRNPC, YME1L1, and OPA1 in hippocampal tissue. *n* = 6. *p < 0.05; **p < 0.01; ****p < 0.0001.

In order to confirm the possible therapeutic benefit of SIRT5 in AD mice, we injected lentiviruses LV‐SIRT5 and LV‐HNRNPC intracerebrally into the APP/PS1 mouse model to overexpress SIRT5 and HNRNPC, respectively. We then assessed the improvement in learning and cognitive abilities as well as the pathology of the brain tissue in AD mice. Behavioral experiments revealed that compared to the WT group, APP/PS1 mice exhibited significant impairments in spatial learning and memory. Compared to the APP/PS1 group, overexpression of SIRT5 significantly improved the spatial learning and memory abilities of mice, as evidenced by a marked increase in platform crossings and time spent in the target quadrant during the water maze test (Figure 9A,B), as well as a shortened escape latency (Figure 9C). In addition, compared with the APP/PS1 group, mice in the LV‐SIRT5 group showed significantly enhanced cognitive function. This was proved by the significantly improved spontaneous alternation rate in the Y‐maze test (Figure 9D), the better object recognition ability in the object recognition test (Figure 9E) and the significantly reduced latency of locating cookies in the olfactory test (Figure 9F). However, the cognitive improvements caused by overexpression of SIRT5 were effectively counteracted by overexpression of HNRNPC (Figure 9A–F), highlighting the neuroprotective effect of SIRT5 depends on its regulation of HNRNPC.

The results of IHC showed that LV‐SIRT5 treatment significantly reduced the deposition of Aβ in the hippocampus, but this effect was also weakened by the overexpression of HNRNPC (Figure 9G). Overexpression of SIRT5 significantly reduced the protein levels of HNRNPC and YME1L1 and improved the hydrolysis balance of OPA1 (increased L‐OPA and decreased S‐OPA1), while overexpression of HNRNPC reversed these changes (Figure 9H). These results indicate that SIRT5 can promote the degradation of HNRNPC by mediating its desuccinylation, then inhibit the expression of YME1L1 and improve mitochondrial function, and finally play a neuroprotective role and improve the cognition and Aβ deposition of AD mice.

## 4 Discussion

Mitochondrial dysfunction is one of the key pathological features of AD, characterized by defects in energy metabolism, excessive ROS production, and mitochondrial dynamics imbalance (Perez Ortiz and Swerdlow 2019; Zhou et al. 2019). Research suggests that posttranslational modifications of proteins, especially succinylation, play a critical regulatory role in various biological metabolic pathways, such as mitochondrial metabolism (Huang et al. 2021; Nita‐Lazar et al. 2021; Yang, Tapias, et al. 2022). In AD, succinylation levels decrease in multiple mitochondrial proteins, while a small number of cytoplasmic proteins show increased succinylation (Yang, Tapias, et al. 2022). Succinylation is regulated by mitochondrial desuccinylase SIRTs, which remove the succinyl group (Yan et al. 2025), but their precise mechanisms in AD remain unclear. Our research shows that the decrease of the expression of SIRT5, a dessuccinylating enzyme, leads to the enhancement of succinylation modification at K50 site of HNRNPC in the pathological environment of AD. This, in turn, promotes the up‐regulation of YME1L1 by stabilizing HNRNPC protein, which eventually leads to the hydrolysis imbalance of OPA1 and the dysfunction of mitochondrial dynamics. Of note, YME1L1 knockdown induced only mild mitochondrial alterations under basal conditions but exhibited relatively more pronounced protective effects under Aβ pathological conditions. This observation is generally consistent with the known role of YME1L1 as a mitochondrial homeostasis‐maintaining protein, while also suggesting that its role is amplified to some extent under AD pathological stress. Based on these findings, YME1L1 not only serves as an important regulator of mitochondrial function but also warrants further exploration as a potential therapeutic target for AD.

Fusion and fission homeostasis represent the most critical components of mitochondrial quality control, also known as mitochondrial dynamics (Koch and Traven 2019). The mitochondrial protease YME1L1 plays a pivotal role in maintaining mitochondrial fusion‐fission balance by regulating OPA1 hydrolysis (Chehaitly et al. 2022; Noone et al. 2022). Our research shows that the expression of YME1L1 is significantly upregulated in APP/PS1 mice and Aβ‐treated neurons. OPA1 is hydrolyzed and regulated by YME1L1, existing in two forms: L‐OPA1 and short S‐OPA1. L‐OPA1 primarily mediates mitochondrial inner membrane fusion, maintaining normal mitochondrial function (Fogo et al. 2024). On the contrary, the accumulation of S‐OPA1 will promote mitochondrial division. We found that in AD, YME1L1 induced mitochondrial disorder by promoting the hydrolysis of OPA1 (leading to the decrease of L‐OPA1 and the accumulation of S‐OPA1). Crucially, the treatment with MYLS22, a specific inhibitor of L‐OPA1, showed that inhibiting the function of L‐OPA1 significantly weakened the protective effect of YME1L1 knockdown, which confirmed that the degradation of OPA1 was the key downstream mechanism of mitochondrial damage mediated by YME1L1. Further research shows that the RNA‐binding protein HNRNPC can improve its stability through m6A modification, thus upregulating YME1L1 mRNA. This discovery provides an important new understanding of the molecular basis of mitochondrial dynamic imbalance and reveals a new way to control the expression of AD gene.

Protein succinylation is a crucial posttranslational modification that not only extensively participates in intracellular metabolic processes but is also closely associated with the onset and progression of numerous metabolic disorders (Wai et al. 2015). Previous studies have demonstrated that abnormal succinylation is associated with AD (Ke et al. 2025). Disruption of APP succinylation impairs its normal proteolytic processing, thereby promoting Aβ accumulation and plaque formation (Yang, Tapias, et al. 2022). Similar to this mechanism, we found that HNRNPC was significantly succinylated in the AD model. Through a site‐mutation experiment, we identified K50 as the key modification site. This modification enhances the stability of the HNRNPC protein by inhibiting the ubiquitin‐proteasome degradation pathway and then regulates the expression of YME1L1. Notably, we found that the K50R mutation significantly enhanced the ubiquitination of HNRNPC, while Co‐IP analysis showed that TRIM25, an E3 ubiquitin ligase, showed stronger binding with the succinylated HNRNPCK50R mutant, indicating that succinylation at the K50 site competitively inhibited TRIM25‐mediated ubiquitination. In addition, blocking succinylation of the K50 site of HNRNPC or inducing K50 site mutations can effectively improve mitochondrial function damage and neuron death, highlighting the therapeutic potential of targeting these modified sites, but overexpression of YME1L1 can abolish the protective effect mediated by HNRNPC^K50R^.

Sirtuin family proteins are NAD+‐dependent deacylases, among which SIRT5, as the primary desuccinylase, plays a crucial role in maintaining mitochondrial protein functional homeostasis (Barreca et al. 2023). Research indicates that SIRT5 regulates the activity and stability of multiple metabolic enzymes, but its role in regulating HNRNPC succinylation in AD remains unclear. Initially, we observed downregulation of SIRT5 expression in AD models, consistent with previous studies (Wu et al. 2021). SIRT5 interacts directly with HNRNPC, which promotes its ubiquitination degradation through succinylation, thus affecting the expression of YME1L1 and the abnormal hydrolysis of OPA1. Overexpression of SIRT5 can reduce the deposition of Aβ, improve mitochondrial function and cognitive impairment by desuccinylating HNRNPC, but overexpression of HNRNPC can reverse this protective effect. In addition, under the background of the HNRNPC K50R mutation, overexpression of SIRT5 could not play a protective role, which completely verified the important role of the SIRT5‐HNRNPC‐YME1L1‐OPA1 regulatory axis in AD.

There are still some limitations in this study. Firstly, the upstream mechanism of downregulation of SIRT5 expression in AD has not been fully revealed. Second, while our K50R mutant experiments provide strong genetic evidence that SIRT5 mediates mitochondrial protection specifically through HNRNPC desuccinylation, we cannot exclude the possibility that other SIRT5 substrates may contribute to additional aspects of mitochondrial function not examined in this study. Third, while SIRT5 overexpression reduced Aβ deposition in AD mice, whether SIRT5 directly regulates APP processing or affects Aβ metabolism through other pathways (such as autophagy or microglial phagocytosis) remains to be investigated in future studies. Finally, the clinical relevance of this mechanism requires confirmation in additional brain tissue samples from AD patients and other AD animal models (such as Tau transgenic mice and sporadic AD models) in future studies.

## 5 Conclusion

In summary, this study demonstrates for the first time that SIRT5‐mediated desuccinylation serves as a critical switch regulating the stability and activity of the HNRNPC protein. In the context of AD, the downregulation of SIRT5 leads to enhanced succinylation of HNRNPC and increased protein stability, which in turn affects the expression of YME1L1 and the abnormal hydrolysis of OPA1, and finally drives mitochondrial fragmentation and dysfunction. Therefore, targeting HNRNPC succinylation‐mediated regulation of YME1L1 expression holds promise as a novel therapeutic strategy for AD.

## Author Contributions

**Xuewei Li:** data curation, formal analysis, funding acquisition, investigation, validation, visualization, writing – original draft, writing – review and editing. **Fan Yang**, **Yuyan Jiang**, **Fei Zhao:** data curation, investigation. **Fan Liu:** conceptualization, data curation, formal analysis, methodology, project administration, resources, supervision, writing – review and editing.

## Funding

This work was supported by the National Key Clinical Specialty Scientific Research Project (grant number: Z2023168).

## Conflicts of Interest

The authors declare no conflicts of interest.

## Supporting information


**Figure S1:** YME1L1 knockdown efficiency validation. (A) PCR and WB were used to detect the expression of YME1L1 in the hippocampus. *n* = 6. ****p < 0.0001.
**Figure S2:** Morphometric quantification of mitochondrial ultrastructure in Figure 1J. (A) Parameters measured include mitochondrial area, perimeter, Feret diameter, aspect ratio, cristae number, and cristae surface area. *n* = 3. ***p < 0.001.
**Figure S3:** Effect of YME1L1 knockdown on mitochondrial function in primary hippocampal neurons under basal conditions. (A) Detection of YME1L1 expression via PCR and WB. (B) CCK8 assay for cell viability. (C) Flow cytometry for apoptosis detection. (D) TEM for mitochondrial assessment (The green arrow indicates normal mitochondria, and the red arrow indicates damaged mitochondria). Scale bar = 500 nm. Parameters measured include mitochondrial damage number, mitochondrial area, perimeter, Feret diameter, aspect ratio, cristae number, and cristae surface area. (E) ATP level measurement. (F) JC‐1 staining for cellular mitochondrial membrane potential measurement. (G) MitoSOX fluorescence staining for cellular mitochondrial ROS analysis. Scale bar = 50 μm. *n* = 3. *p < 0.05; **p < 0.01; ***p < 0.001; ****p < 0.0001.
**Figure S4:** Morphometric quantification of mitochondrial ultrastructure in Figure 2C (A) and 3E (B). Parameters measured include mitochondrial area, perimeter, Feret diameter, aspect ratio, cristae number, and cristae surface area. *n* = 3. *p < 0.05; **p < 0.01; ***p < 0.001.
**Figure S5:** S‐OPA1 overexpression reverses the protective effects of YME1L1 knockdown against Aβ1‐42‐induced neuronal injury. (A) Detection of OMA1 expression via WB. *n* = 6. (B) CCK8 assay for cell viability. (C) Flow cytometry for apoptosis detection. (D) TEM for mitochondrial assessment (The green arrow indicates normal mitochondria, and the red arrow indicates damaged mitochondria). Scale bar = 500 nm. Parameters measured include mitochondrial damage number, mitochondrial area, perimeter, Feret diameter, aspect ratio, cristae number, and cristae surface area. (E) ATP level measurement. (F) JC‐1 staining for cellular mitochondrial membrane potential measurement. (G) MitoSOX fluorescence staining for cellular mitochondrial ROS analysis. Scale bar = 50 μm. *n* = 3. *p < 0.05; **p < 0.01; ***p < 0.001.
**Figure S6:** Upregulation of HNRNPC in AD models and validation of HNRNPC siRNA knockdown efficiency. (A) WB detection of HNRNPC expression in hippocampal tissue. *n* = 6. (B) WB detection of HNRNPC expression. (C) PCR and WB detection of HNRNPC expression. *n* = 3. **p < 0.01; ****p < 0.0001.
**Figure S7:** Morphometric quantification of mitochondrial ultrastructure in Figure 6D. (A) Parameters measured include mitochondrial area, perimeter, Feret diameter, aspect ratio, cristae number, and cristae surface area. *n* = 3. **p < 0.01; ***p < 0.001.
**Figure S8:** Downregulation of SIRT5 and SIRT7 in AD models and validation of SIRT5 and SIRT7 overexpression efficiency. (A) WB detection of SIRT5 and SIRT7 expression in hippocampal tissue. *n* = 6. (B) WB detection of SIRT5 and SIRT7 expression. (C) WB detection of SIRT5 expression. (D) WB detection of SIRT7 expression. *n* = 3. *p < 0.05; ***p < 0.001; ****p < 0.0001.
**Figure S9:** Morphometric quantification of mitochondrial ultrastructure in Figure 8D. (A) Parameters measured include mitochondrial area, perimeter, Feret diameter, aspect ratio, cristae number, and cristae surface area. *n* = 3. ***p < 0.001; ns stands for no significant difference.

## Data Availability

The datasets generated during and analyzed during the current study are not publicly available but are available from the corresponding author on reasonable request.
