# Supplementary material for: HNRNPC Succinylation Influences the Neurodegeneration of Alzheimer's Disease Through YME1L1‐Mediated Mitochondrial Metabolism
Source: Aging Cell. 2026 Aug 5;25(8):e70646. doi: 10.1111/acel.70646 (PMC13443708; doi:10.1111/acel.70646)
Supplement: Supplementary file 1 [file ACEL-25-e70646-s001.docx]

**Figs.S1-S9**

**Supplementary Figure S1. YME1L1 knockdown efficiency validation. (**A) PCR and WB were used to detect the expression of YME1L1 in the hippocampus. n=6. *****P* < 0.0001.

**Supplementary Figure S2. Morphometric quantification of mitochondrial ultrastructure in Figure 1J.** (A) Parameters measured include mitochondrial area, perimeter, Feret diameter, aspect ratio, cristae number, and cristae surface area. n=3. ****P* < 0.001.

**Supplementary Figure S3. Effect of YME1L1 knockdown on mitochondrial function in primary hippocampal neurons under basal conditions.** (A) Detection of YME1L1 expression via PCR and WB. (B) CCK8 assay for cell viability. (C) Flow cytometry for apoptosis detection. (D) TEM for mitochondrial assessment (The green arrow indicates normal mitochondria, and the red arrow indicates damaged mitochondria). Scale bar = 500 nm. Parameters measured include mitochondrial damage number, mitochondrial area, perimeter, Feret diameter, aspect ratio, cristae number, and cristae surface area. (E) ATP level measurement. (F) JC-1 staining for cellular mitochondrial membrane potential measurement. (G) MitoSOX fluorescence staining for cellular mitochondrial ROS analysis. Scale bar = 50 μm. n=3. **P* < 0.05; ***P* < 0.01; ****P* < 0.001; *****P* < 0.0001.

**Supplementary Figure S4. Morphometric quantification of mitochondrial ultrastructure in Figure 2C (A) and 3E (B).** Parameters measured include mitochondrial area, perimeter, Feret diameter, aspect ratio, cristae number, and cristae surface area. n=3. **P* < 0.05; ***P* < 0.01; ****P* < 0.001.

**Supplementary Figure S5. S-OPA1 overexpression reverses the protective effects of YME1L1 knockdown against Aβ1-42-induced neuronal injury.** (A) Detection of OMA1 expression via WB. n=6. (B) CCK8 assay for cell viability. (C) Flow cytometry for apoptosis detection. (D) TEM for mitochondrial assessment (The green arrow indicates normal mitochondria, and the red arrow indicates damaged mitochondria). Scale bar = 500 nm. Parameters measured include mitochondrial damage number, mitochondrial area, perimeter, Feret diameter, aspect ratio, cristae number, and cristae surface area. (E) ATP level measurement. (F) JC-1 staining for cellular mitochondrial membrane potential measurement. (G) MitoSOX fluorescence staining for cellular mitochondrial ROS analysis. Scale bar = 50 μm. n=3. **P* < 0.05; ***P* < 0.01; ****P* < 0.001.

**Supplementary Figure S6. Upregulation of HNRNPC in AD models and validation of HNRNPC siRNA knockdown efficiency.** (A) WB detection of HNRNPC expression in hippocampal tissue. n=6. (B) WB detection of HNRNPC expression. (C) PCR and WB detection of HNRNPC expression. n=3. ***P* < 0.01; *****P* < 0.0001.

**Supplementary Figure S7. Morphometric quantification of mitochondrial ultrastructure in Figure 6D.** (A) Parameters measured include mitochondrial area, perimeter, Feret diameter, aspect ratio, cristae number, and cristae surface area. n=3. ***P* < 0.01; ****P* < 0.001.

**Supplementary Figure S8. Downregulation of SIRT5 and SIRT7 in AD models and validation of SIRT5 and SIRT7 overexpression efficiency.** (A) WB detection of SIRT5 and SIRT7 expression in hippocampal tissue. n=6. (B) WB detection of SIRT5 and SIRT7 expression. (C) WB detection of SIRT5 expression. (D) WB detection of SIRT7 expression. n=3. **P* < 0.05; ****P* < 0.001; *****P* < 0.0001.

**Supplementary Figure S9. Morphometric quantification of mitochondrial ultrastructure in Figure 8D.** (A) Parameters measured include mitochondrial area, perimeter, Feret diameter, aspect ratio, cristae number, and cristae surface area. n=3. ****P* < 0.001; ns stands for no significant difference.
